# Comparative Circulating Interleukin‐6 Levels in Human and Mouse Periodontitis: A Systematic Review and Meta‐Analysis

**DOI:** 10.1111/odi.70367

**Published:** 2026-05-20

**Authors:** Jeong‐Min Lee, Kyeongho Lee, June Yeon Kim, Hun Jin Jeong, Sung‐Jin Kim

**Affiliations:** ^1^ Department of Oral Histology and Developmental Biology, School of Dentistry and Dental Research Institute Seoul National University Seoul Korea; ^2^ Department of Engineering University of Maryland Eastern Shore Princess Anne Maryland USA

**Keywords:** interleukin‐6, meta‐analysis, periodontitis, systemic inflammation

## Abstract

**Objective:**

To quantitatively compare circulating interleukin‐6 (IL‐6) levels in human periodontitis and mouse models, and to evaluate the impact of experimental induction methods on systemic inflammatory responses.

**Methods:**

A systematic review and meta‐analysis was conducted according to PRISMA guidelines. Studies reporting serum or plasma IL‐6 levels in periodontitis and controls were included. Human studies involved Stage III or higher periodontitis; mouse studies used ligation or inoculation models. Standardized mean differences (SMDs) were pooled using random‐effects models.

**Results:**

Thirty‐eight studies (26 human, 12 mouse) were included. Periodontitis was associated with elevated circulating IL‐6 in humans (SMD = 1.57; 95% CI: 0.61–2.54) and mice (SMD = 3.79; 95% CI: 2.53–5.05), with greater increases in mice (*p* = 0.006). Ligature‐induced models showed the largest elevation (SMD = 4.39), higher than humans (*p* = 0.002) and inoculation models (*p* = 0.006), whereas inoculation models did not differ from humans. Meta‐regression confirmed stronger IL‐6 responses in mice (*β* = 2.26; *p* = 0.007) and ligature models (*β* = 2.85; *p* = 0.006), indicating amplified responses in ligature‐induced models.

**Conclusions:**

Circulating IL‐6 is elevated in periodontitis across species but exaggerated in ligation models, suggesting possible overestimation of systemic inflammation in preclinical studies.

**Trial Registration:**

PROSPERO: CRD420251101961

## 1 Introduction

Periodontitis is a chronic inflammatory disease characterized by the progressive destruction of the tooth‐supporting tissues and is a major cause of tooth loss in adults (Papapanou et al. 2018a). Beyond its local effects, epidemiological studies have reported associations between periodontitis and various systemic conditions, including cardiovascular disease, diabetes mellitus, and chronic obstructive pulmonary disease (Nabila et al. 2023; Sczepanik et al. 2020). However, the observational design of these studies precludes definitive conclusions regarding causality.

To address causality, experimental mouse models have been widely used, employing ligature placement or oral inoculation with periodontopathogenic bacteria (Abe and Hajishengallis 2013; Hernandez Martinez et al. 2023). These models demonstrate that periodontitis elevates circulating inflammatory mediators, including interleukin‐6 (IL‐6), tumor necrosis factor‐α (TNF‐α), and C‐reactive protein (CRP), and may exacerbate systemic comorbidities (Kashiwagi et al. 2021; Li et al. 2024; Maresz et al. 2013; Suh et al. 2019). However, they differ substantially from human disease in time course, microbial complexity, and host characteristics, raising concerns that systemic effects may be exaggerated or distorted (Rojas et al. 2021). In addition, recent studies have highlighted that periodontal treatment, including antimicrobial interventions, may modulate both local and systemic inflammatory responses, further emphasizing the clinical relevance of accurately interpreting inflammatory changes in periodontitis (Isola et al. 2026; Rocco et al. 2025).

Among inflammatory mediators, IL‐6—a pleiotropic pro‐inflammatory cytokine—has received particular attention as a central marker of systemic inflammation (Rose‐John 2022). Circulating IL‐6 is consistently associated with cardiovascular disease, type 2 diabetes, and rheumatoid arthritis, and elevated levels have been reported in both human periodontitis and experimental mouse models (Kashiwagi et al. 2021; Maresz et al. 2013; Suh et al. 2019). These observations establish circulating IL‐6 as a biologically meaningful and clinically relevant marker for evaluating the systemic impact of periodontitis across experimental models and human disease.

To date, no systematic review or meta‐analysis has quantitatively compared circulating IL‐6 responses between humans and mice with periodontitis, nor assessed the impact of induction methods. Given the widespread use of experimental models to infer systemic effects of periodontitis, a direct comparison of inflammatory responses across species is critical for evaluating their translational validity. Therefore, we conducted a systematic review and meta‐analysis to evaluate serum or plasma IL‐6 levels, quantify interspecies differences, and examine the influence of induction models.

## 2 Materials and Methods

This systematic review and meta‐analysis was conducted in accordance with the Preferred Reporting Items for Systematic Reviews and Meta‐Analyses (PRISMA) guidelines (Page et al. 2021). Protocol was registered in the International Prospective Register of Systematic Reviews (PROSPERO).

### 2.1 Focused Question

The focused question was formulated using the PECO framework to evaluate whether periodontitis leads to comparable changes in circulating IL‐6 levels between humans and mouse models when each is compared to its respective healthy controls. Specifically, the question addressed was: “In both humans and mouse models, does the presence of periodontitis lead to comparable changes in serum or plasma IL‐6 levels, used as an indicator of systemic inflammation, when compared to their respective healthy controls?”

Population (P): Human patients with periodontitis and mice with experimentally induced periodontitis.

Exposure (E): Presence of periodontitis (naturally occurring in humans, experimentally induced in mice).

Comparator (C): Periodontally healthy humans and mice as control groups.

Outcome (O): Serum or plasma IL‐6 levels as an indicator of systemic inflammation.

### 2.2 Search Strategy

An electronic literature search was conducted using PubMed, Scopus, Embase, and Web of Science to identify studies published up to May 2025. The search was restricted to articles published in English.

The search strategy combined Title/Abstract keywords related to periodontitis, circulating IL‐6, and sample type, as follows:

PubMed:

- (“Periodontitis”[Title/Abstract] OR “Periodontal Disease”[Title/Abstract]) AND (“Blood”[Title/Abstract] OR “Serum”[Title/Abstract] OR “Plasma”[Title/Abstract]) AND (“Interleukin”[Title/Abstract] OR “Interleukin‐6”[Title/Abstract] OR “IL‐6”[Title/Abstract] OR “Cytokines”[Title/Abstract]) AND (“Mouse”[Title/Abstract] OR “Mice”[Title/Abstract] OR “Human”[Title/Abstract] OR “Patients”[Title/Abstract] OR “Cohort”[Title/Abstract] OR “Animal Model”[Title/Abstract])

Scopus:

- TITLE‐ABS‐KEY ((periodontitis OR “periodontal disease”) AND (“interleukin‐6” OR “IL‐6” OR interleukin) AND (blood OR serum OR plasma) AND (human OR patient* OR mouse OR mice)) AND PUBYEAR > 1982 AND PUBYEAR < 2026 AND (LIMIT‐TO (SRCTYPE, “j”)) AND (EXCLUDE (DOCTYPE, “re”)) AND (LIMIT‐TO (LANGUAGE, “English”))

Web of Science:

- TS = ((periodontitis OR “periodontal disease”) AND (“interleukin‐6” OR “IL‐6” OR interleukin) AND (blood OR serum OR plasma) AND (human OR patient* OR mouse OR mice))

Embase:

- (“periodontitis”/exp OR periodontitis:ti,ab OR “periodontal disease”:ti,ab) AND (“interleukin 6”/exp OR “interleukin 6”:ti,ab OR “il 6”:ti,ab OR interleukin:ti,ab) AND (“blood”/exp OR blood:ti,ab OR serum:ti,ab OR plasma:ti,ab) AND (“human”/exp OR “mouse”/exp OR human:ti,ab OR patient*:ti,ab OR mouse:ti,ab OR mice:ti,ab)

The literature search results were imported into ASReview (ASReview LAB, Utrecht University, the Netherlands) for duplicate removal and machine learning–assisted title and abstract screening. The screening process was conducted using an active learning approach to prioritize relevant studies.

### 2.3 Study Selection

We selected original research articles according to the following inclusion criteria. For human studies, we included studies reporting serum or plasma IL‐6 concentrations in adults with periodontitis of Stage III or higher, along with a periodontally healthy control group. For animal studies, we included studies using mouse models of experimental periodontitis induced by ligature placement or oral bacterial inoculation, with a corresponding healthy control group. Only articles published in English were considered.

Studies were excluded if they: (i) included participants with systemic diseases such as diabetes or cardiovascular disease, to minimize confounding effects on circulating IL‐6 levels; (ii) lacked a periodontally healthy control group; (iii) were in vitro studies, reviews, case reports, or letters; (iv) did not report quantitative IL‐6 data as mean and standard deviation (SD) or lacked sufficient information for conversion; (v) did not provide sufficient information to determine periodontitis stage; or (vi) included only aggressive or acute forms of periodontitis.

Given the variability in diagnostic criteria for periodontitis across studies, we included studies that met at least one of the following: (1) explicit classification as Stage III or IV periodontitis according to the 2018 AAP/EFP case definition (Papapanou et al. 2018b); or (2) sufficient clinical parameters, such as clinical attachment level, probing depth, or radiographic bone loss, to permit retrospective staging or classification according to these systems.

Following the eligibility criteria, two reviewers (J.‐M.L. and K.L.) independently screened the titles and abstracts of all identified studies to exclude irrelevant articles, followed by full‐text assessment to determine final eligibility. Disagreements were resolved through discussion.

### 2.4 Data Extraction

After study selection, two reviewers independently performed data extraction using a standardized Excel spreadsheet. One reviewer extracted all relevant data, and a second reviewer checked the extracted data for accuracy and completeness. Prior to formal data extraction, the Excel form was piloted on a small subset of studies to ensure that all necessary variables were captured consistently.

The following variables were extracted from each included study. For human studies, extracted data included study characteristics, participant characteristics, and IL‐6 concentration (mean and SD). For mouse studies, extracted data included study characteristics, model type, induction method, induction period, periodontitis evaluation method, blood collection type, and IL‐6 concentration data. When multiple independent experiments were reported in the same paper, each was treated as a separate study. For both human and mouse studies, when IL‐6 concentration data were reported as medians with interquartile ranges or ranges, values were converted to means and SDs using established methods (Luo et al. 2018; Wan et al. 2014).

### 2.5 Risk of Bias Assessment

The risk of bias for included human studies was assessed using the ROBINS‐E tool (Higgins et al. 2024), which evaluates bias domains related to confounding, participant selection, classification of exposures, deviations from intended exposures, missing data, measurement of outcomes, and selection of the reported result. For mouse studies, the SYRCLE's risk of bias tool (Hooijmans et al. 2014) was used to assess methodological quality, including selection, performance, detection, attrition, and reporting biases across 10 domains. Risk of bias assessment figures were generated using R software (version 2025.05.0+496, R Foundation for Statistical Computing, Vienna, Austria), with traffic light plots created using the robvis package (McGuinness and Higgins 2021) and summary bar plots generated using ggplot2 (Wickham 2016).

Two reviewers independently performed the risk of bias assessments for both human and mouse studies. Any disagreements were resolved through discussion to reach consensus. The overall risk of bias for each mouse study was determined as high if any domain was judged high risk, unclear if at least one domain was unclear and nonhigh risk, and low if all domains were low risk.

### 2.6 Statistical Analysis

Meta‐analyses were conducted to estimate the standardized mean differences (SMD) in serum or plasma IL‐6 levels between periodontitis groups and their respective healthy control groups. For each study, SMDs with 95% confidence intervals (CIs) were calculated and pooled using a random‐effects model (Restricted Maximum Likelihood method) to account for heterogeneity across studies. The pooled effect was considered statistically significant if two‐sided *p*‐values were < 0.05. Heterogeneity was assessed using Cochran's *Q* test and quantified with the *I* ^2^ statistic, with *I* ^2^ values of 25%, 50%, and 75% considered to represent low, moderate, and high heterogeneity, respectively.

To explore differences across species and induction methods, subgroup analyses were conducted using random‐effects models. Comparisons between humans and mice were assessed with *z*‐tests, and meta‐regression with species as a moderator was applied to confirm interspecies contrasts. For analyses involving humans, ligature‐ and inoculation‐induced models, heterogeneity among the three groups was evaluated using *Q*‐test, followed by pairwise subgroup comparisons with *z*‐tests. Meta‐regression was further performed with group as a categorical moderator, reporting regression coefficients (*β*) with 95% CI to quantify group‐level differences. For these regression‐based contrasts, *p*‐values were adjusted for multiple testing using the Holm method.

Publication bias was analyzed using funnel plots and Egger's statistical test. Sensitivity analyses were conducted using the Leave‐One‐Out method, whereby each study was sequentially removed and the pooled estimate recalculated to evaluate the influence of individual studies on the overall effect size.

All statistical analyses were performed using R software (version 2025.05.0+496, R Foundation for Statistical Computing, Vienna, Austria) with the metafor package (Viechtbauer and Cheung 2010) for effect‐size estimation, and the multcomp package for linear contrasts and multiple comparisons in meta‐regression. Forest plots were generated to visualize the pooled effect sizes.

## 3 Results

### 3.1 Study Selection

Figure 1 displays the flow chart of the study selection process. A total of 7474 records were identified through the PubMed database. After removing one duplicate, 2900 records were screened, and 113 full‐text articles were assessed for eligibility. Thirty‐eight studies met the inclusion criteria and were included in the systematic review. Details of excluded studies and reasons for exclusion are presented in Table S1.

**FIGURE 1:**
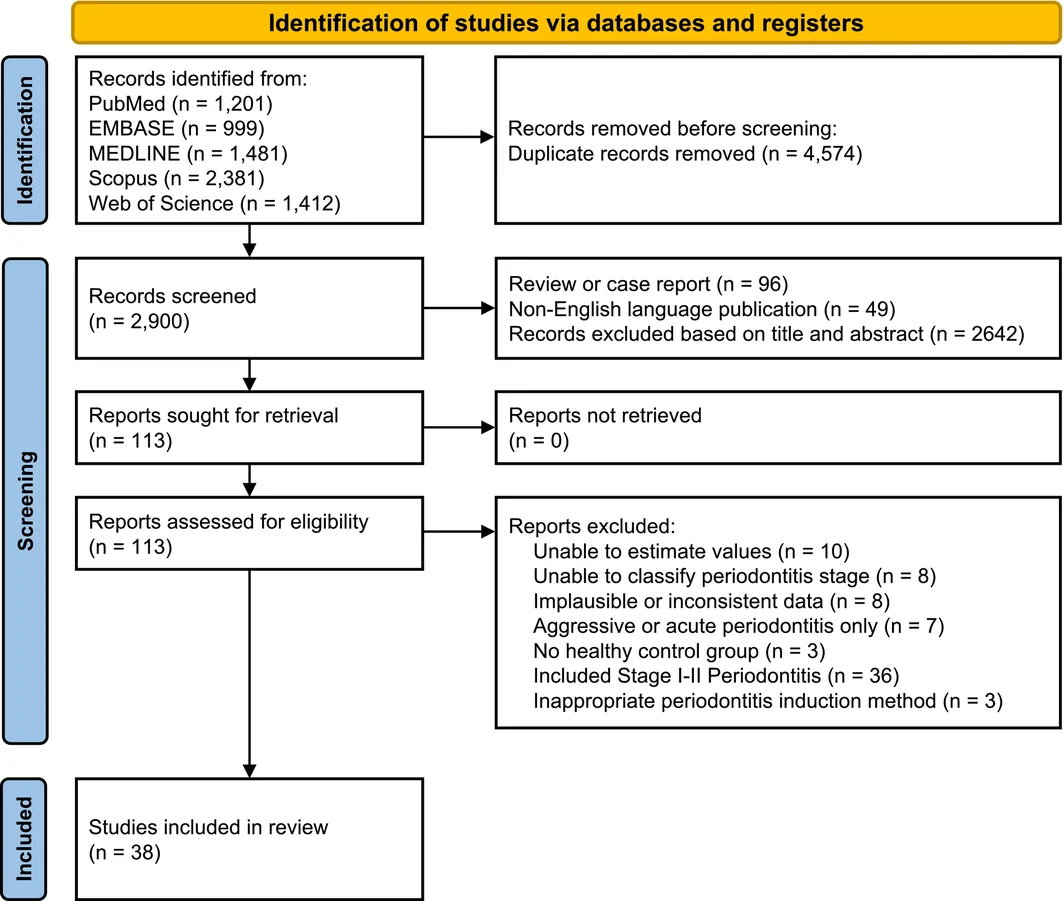
PRISMA 2020 flow chart of study selection process. For details regarding reasons for exclusion from meta‐analysis, see Table S1.

### 3.2 Study Characteristics

The characteristics of the included studies are summarized in Tables S2 and S3. A total of 26 human studies and 12 mouse studies were included in this systematic review, comprising 26 and 14 datasets, respectively. Among the human studies, 15 (57.7%) were case–control, 9 (34.6%) were cross‐sectional, and 2 (7.7%) were intervention in design. Studies were conducted across 10 countries, and sample sizes ranged from 30 to 1290 participants. Mean age ranged from 32.7 to 72.7 years, and male proportion varied from 26.9% to 62.6%. Fasting status was reported in 9 studies, and BMI control was performed in 8 studies. Serum was the most commonly used biospecimen. All human studies involved participants with periodontitis equivalent to Stage III and above based on the 2017 AAP/EFP classification system. Periodontitis diagnostic criteria were reclassified when necessary, based on clinical probing depth, attachment loss, and radiographic bone loss, and were considered secure.

Among mouse studies, 9 used ligature‐induced models and 3 used oral inoculation models; all studies used C57BL/6 mice, predominantly male, and employed induction periods ranging from 7 days to 12 months. Periodontitis was assessed by CEJ‐ABC distance, histological evaluation, and/or gingival cytokine analysis.

### 3.3 Risk of Bias

In human studies, most were judged to have low or moderate risk of bias. Bias due to confounding and selection into the study were the most frequently identified concerns, while measurement of the exposure, outcome assessment, and missing data were generally well addressed. A few studies showed high risk in the domains of selective reporting and confounding (Figure 2A).

**FIGURE 2:**
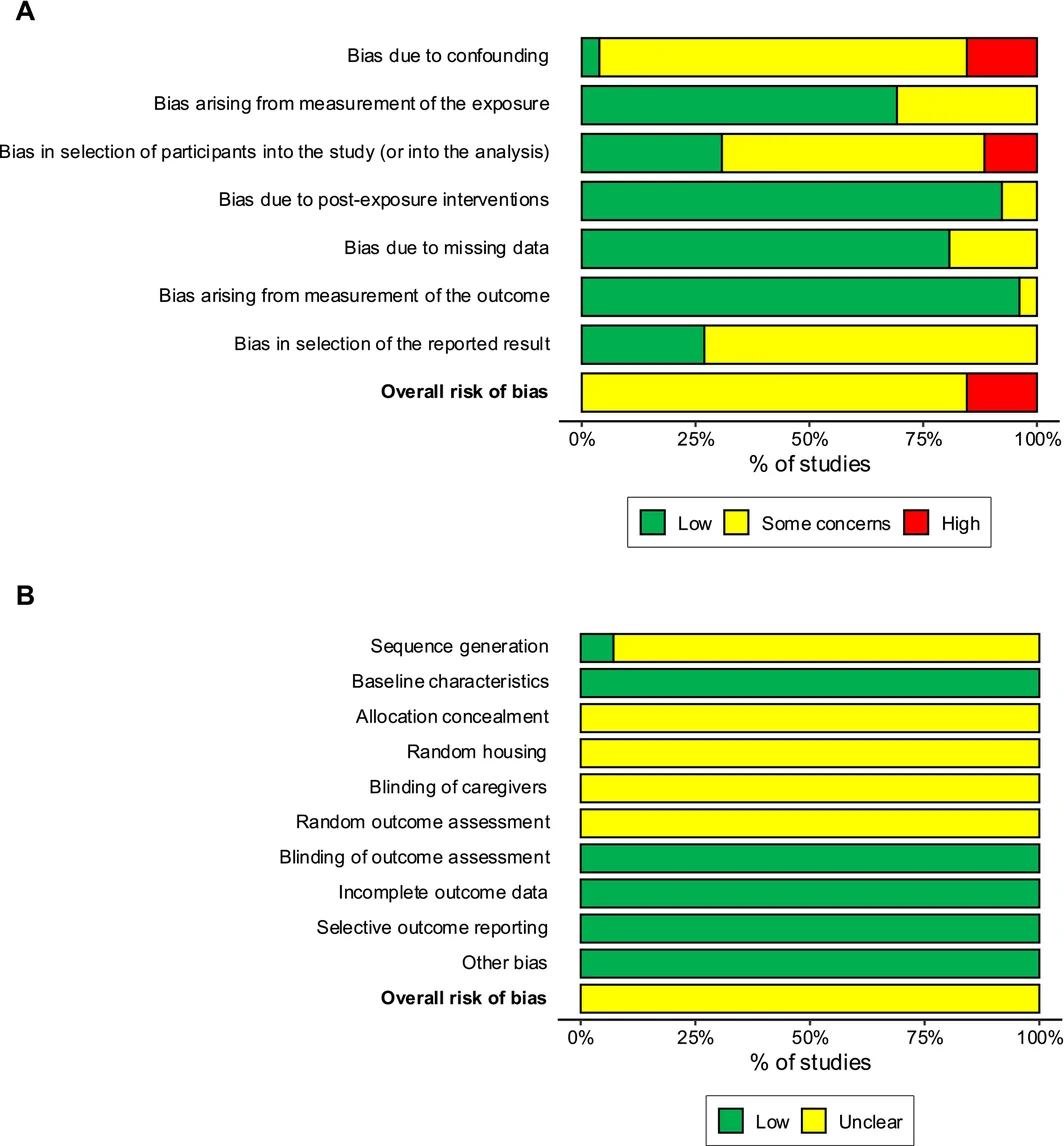
Risk of bias assessment of included studies. (A) Human studies assessed with the ROBINS‐E tool. (B) Mouse studies assessed with the SYRCLE risk of bias tool. For full domain‐level assessments for all individual studies, see Figures S1 and S2.

In the mouse studies, outcome‐related domains such as blinding and completeness of outcome data were generally assessed as low risk. In contrast, methodological domains including allocation concealment, random housing, and sequence generation were frequently rated as unclear due to insufficient reporting. No studies were judged to have high risk in any domain (Figure 2B). The detailed domain‐level risk of bias assessments for individual studies are presented in a traffic‐light plot (Figures S1 and S2).

### 3.4 Association Between Periodontitis and Circulating IL‐6 Level

A total of 26 human studies (26 datasets) and 12 mouse studies (14 datasets) were included in the meta‐analysis comparing circulating IL‐6 levels between periodontitis and control groups. In human studies, the pooled effect estimate indicated significantly higher IL‐6 levels in periodontitis patients (SMD: 1.57; 95% CI: 0.61–2.54; *p* = 0.001; *I* ^2^ = 99.1%) (Figure 3A). In mouse models, the pooled effect estimates also demonstrated higher IL‐6 levels in the periodontitis group than in controls (SMD: 3.79; 95% CI: 2.53–5.05; *p* < 0.001; *I* ^2^ = 88.1%) (Figure 3B). Both human and mouse studies showed wide prediction intervals, indicating substantial between‐study variability.

**FIGURE 3:**
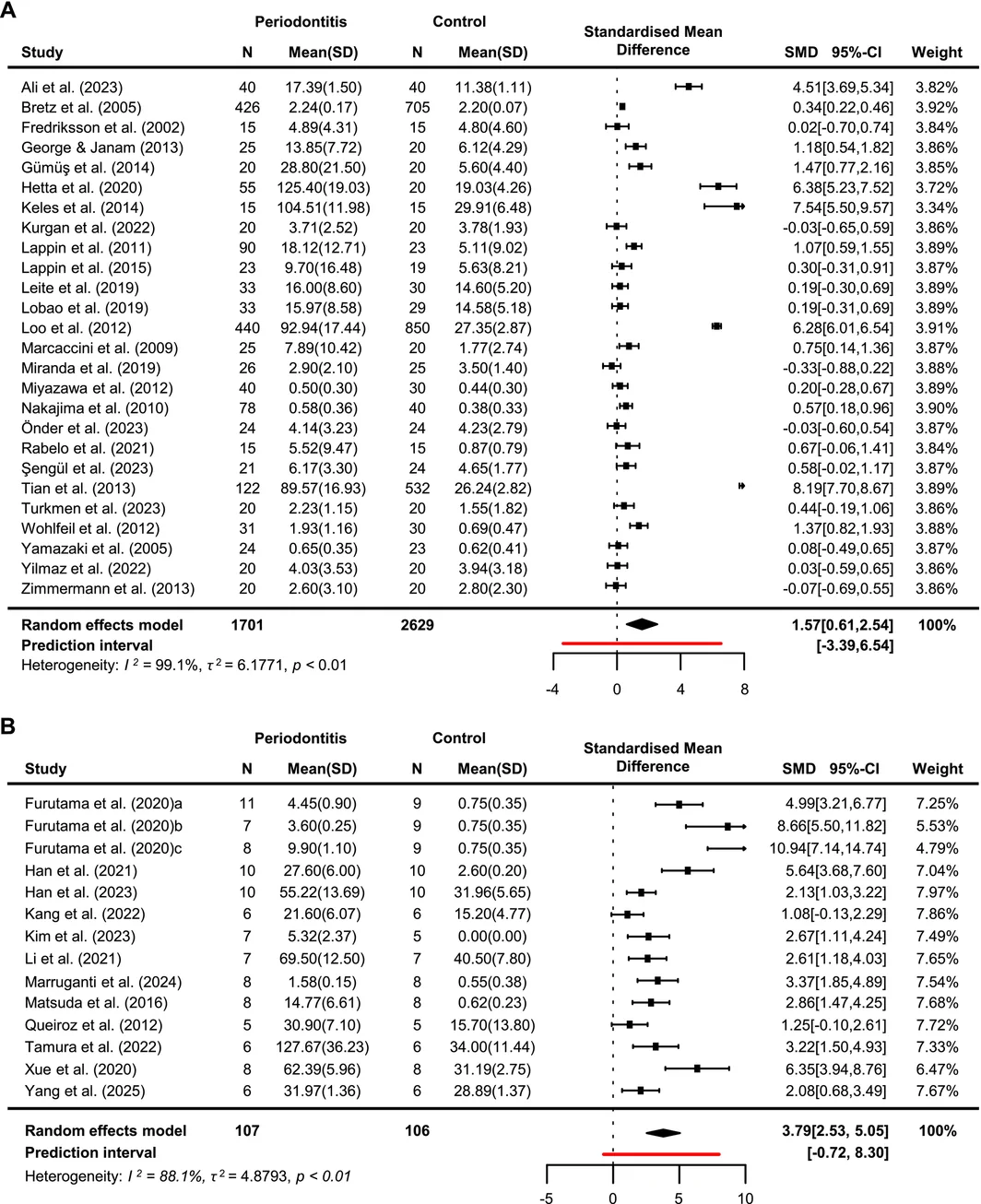
Forest plots of circulating IL‐6 levels in periodontitis compared with controls. (A) Human studies (*n* = 26 datasets). (B) Mouse studies (*n* = 14 datasets, with datasets from one study denoted as a, b, and c when different experimental conditions were reported). Effect sizes are presented as standardized mean differences (SMD) with 95% confidence intervals (CI), estimated using random‐effects models. Squares indicate individual study estimates (size proportional to study weight), horizontal lines represent 95% CI, and diamonds denote pooled effect estimates. Heterogeneity across studies is summarized by the *I* ^2^ statistic. Red lines represent 95% prediction intervals.

When mouse studies were stratified by induction method, ligature‐induced models (Figure 4A) showed markedly elevated IL‐6 levels compared with controls (SMD: 4.39; 95% CI: 2.93–5.86; *p* < 0.001; *I* ^2^ = 87.7%), with a wide prediction interval (−0.28 to 9.07) reflecting substantial heterogeneity. Inoculation‐induced models (Figure 4B) also demonstrated significantly higher IL‐6 levels in periodontitis groups (SMD: 1.73; 95% CI: 0.51–2.95; *p* = 0.005; *I* ^2^ = 55.2%), although this finding was based on three studies. Overall, all groups—including human studies and both ligature‐ and inoculation‐induced subgroups—showed significantly elevated circulating IL‐6 levels in periodontitis compared with controls.

**FIGURE 4:**
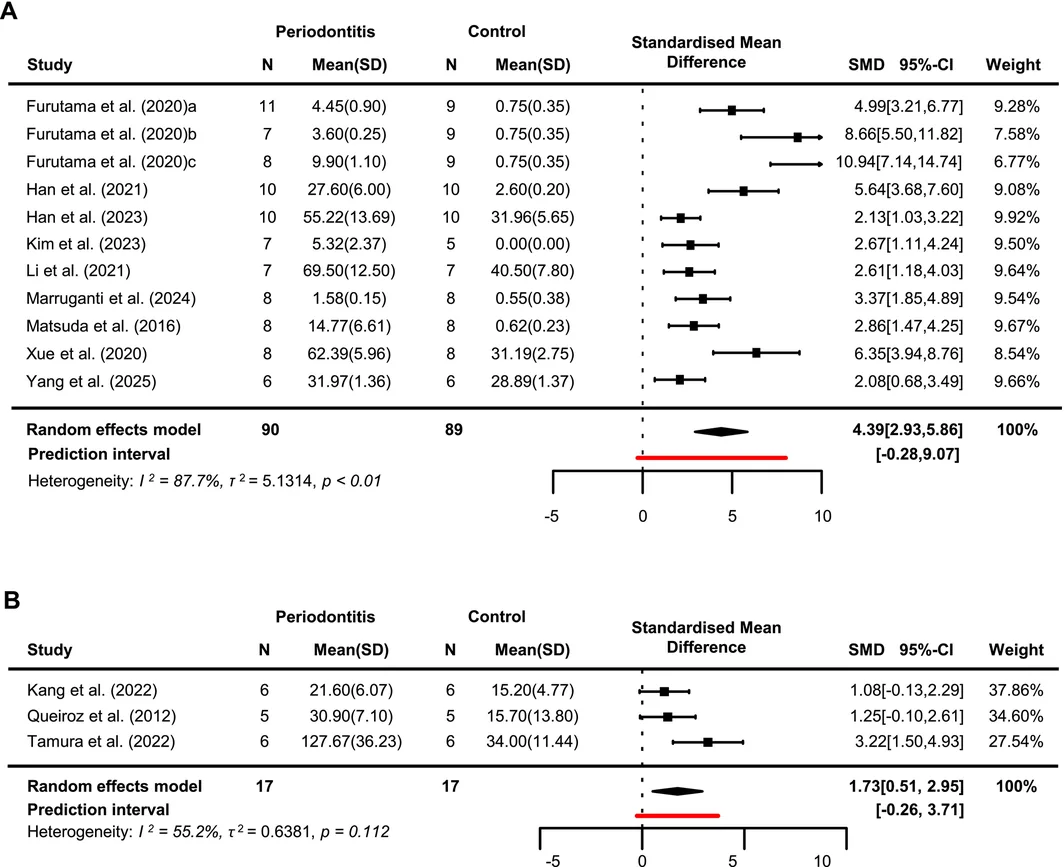
Forest plots of circulating IL‐6 levels in mouse periodontitis models stratified by induction method. (A) Ligature‐induced models (*n* = 11 datasets, with datasets from one study denoted as a, b, and c when different experimental conditions were reported). (B) Inoculation‐induced models (*n* = 3 datasets). Effect sizes are presented as standardized mean differences (SMD) with 95% confidence intervals (CI), estimated using random‐effects models. Squares indicate individual study estimates (size proportional to study weight), horizontal lines represent 95% CI, and diamonds denote pooled effect estimates. Heterogeneity across studies is summarized by the *I* ^2^ statistic. Red lines represent 95% prediction intervals.

### 3.5 Comparisons Across Species and Experimental Models

To further assess differences across species and experimental models, subgroup analyses and meta‐regression were conducted to compare the magnitude of IL‐6 elevation between human and mouse studies, as well as between ligature‐ and inoculation‐induced mouse models. In the comparison of human and mouse studies, subgroup analysis revealed significantly greater IL‐6 elevation in mice compared with humans (*p* = 0.006). Meta‐regression with species as a moderator confirmed this finding, showing higher IL‐6 elevation in mouse studies than in human studies (*β* = 2.26; 95% CI: 0.60–3.92; *p* = 0.007) (Figure 5A).

**FIGURE 5:**
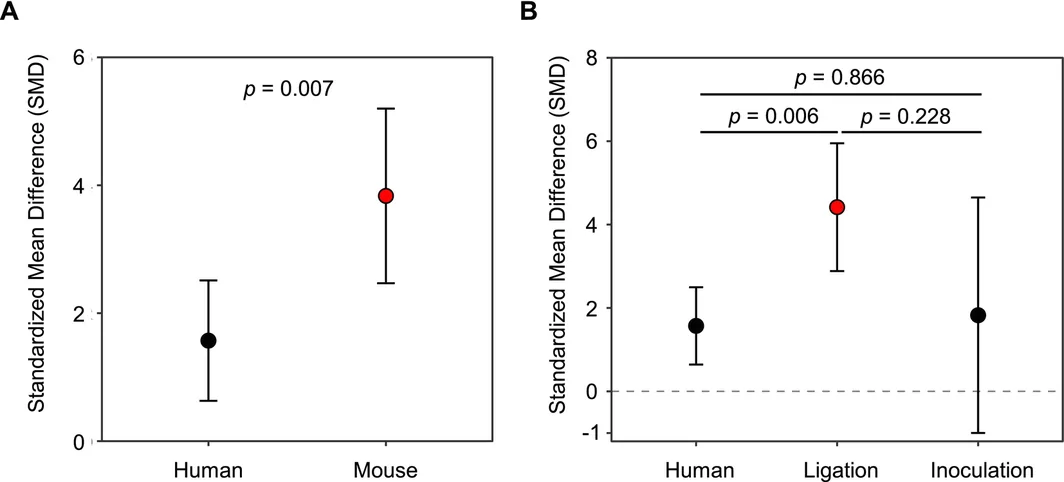
Meta‐regression analyses of circulating IL‐6 responses in humans and mouse periodontitis models. (A) Meta‐regression comparing standardized mean differences (SMD) in circulating IL‐6 levels between humans and mice with periodontitis relative to healthy controls. (B) Meta‐regression including induction method as a categorical moderator. Points indicate pooled effect estimates, and vertical bars represent 95% confidence intervals (CI). *p*‐values represent statistical significance of between‐group comparisons.

When mouse studies were stratified by induction method, subgroup analysis indicated significant heterogeneity among humans, ligature models, and inoculation models (*p* < 0.01). Pairwise comparisons showed that ligature models had significantly larger effects than both humans (*p* = 0.002) and inoculation models (*p* = 0.006), whereas humans and inoculation models did not differ significantly. These results were corroborated by meta‐regression with group as a categorical moderator: compared with humans, ligature models showed a significantly greater effect (*β* = 2.85; 95% CI: 1.06–4.64; *p* = 0.006), whereas inoculation models did not (*β* = 0.26; 95% CI: −2.72 to 3.23; *p* = 0.866). Ligature models also tended to differ from inoculation models (*β* = 2.59; 95% CI: −0.62 to 5.80; *p* = 0.228), although this difference did not reach statistical significance (Figure 5B). Collectively, these analyses indicate that the IL‐6 response to periodontitis is greater in mice than in humans, particularly in ligature‐induced models.

### 3.6 Sensitivity Analysis and Publication Bias

To ensure that the meta‐analysis results were not unduly influenced by any single study, we performed a sensitivity analysis in which each study was sequentially excluded to assess its impact on the overall effect estimates. No significant changes in the magnitude or direction of the effect sizes were observed in either the human or mouse analyses, confirming the stability of the findings (Figure S3).

Publication bias was assessed using funnel plot asymmetry and Egger's regression test. Egger's regression test indicated no significant asymmetry in the human studies (*p* = 0.541) (Figure S4A) and inoculation‐induced mouse studies (*p* = 0.159) (Figure S4B), whereas significant asymmetry was observed in both the all mouse studies (*p* < 0.001) (Figure S4C) and ligature‐induced mouse studies (*p* < 0.001) (Figure S4D). To further evaluate the potential impact of publication bias, the trim‐and‐fill method was applied to the all mouse and ligature‐induced mouse analyses. In both cases, no missing studies were imputed, and the adjusted effect sizes (all mouse: SMD: 3.79, 95% CI: 2.53–5.05; ligature‐induced mouse: SMD: 4.40, 95% CI: 2.93–5.86) were identical to the original estimates, indicating that the presence of publication bias is unlikely to have materially influenced the pooled results.

## 4 Discussion

This systematic review and meta‐analysis evaluated the association between periodontitis and circulating IL‐6 levels in both human populations and mouse models. To our knowledge, it is the first meta‐analysis to quantitatively compare IL‐6 responses between humans and mice in this context. Across 38 eligible studies, circulating IL‐6 levels were generally higher in periodontitis than controls. To account for species‐specific baseline and assay differences, we expressed IL‐6 responses in both humans and mice as standardized mean differences (SMD) between periodontitis and control groups, enabling direct cross‐species comparison. Effect sizes were substantially larger in mouse models—particularly in those employing ligature‐induced periodontitis—than in human studies. Overall, these results indicate that experimental models, especially ligature‐induced models, exhibit amplified systemic IL‐6 responses compared to human periodontitis. These findings support the biological plausibility that periodontitis contributes to systemic inflammation (D'Aiuto et al. 2010; Loos 2005), while highlighting the importance of critically considering methodological differences between human and animal studies when interpreting inflammatory biomarkers.

Periodontitis is characterized by a dynamic inflammatory milieu in which multiple biomarkers, including IL‐6, TNF‐α, and CRP, evolve with disease progression and severity, reflecting the host response to microbial dysbiosis (Isola et al. 2023). Consistent with this, periodontitis has been linked to elevated systemic levels of these inflammatory mediators (Jain et al. 2020; Loos 2005; Loos et al. 2000; Machado et al. 2021). Among these, IL‐6 was selected as a surrogate marker in this study, given its well‐established role in systemic inflammation and its consistent association with cardiometabolic and autoimmune diseases (Danesh et al. 2008; Kaptoge et al. 2014; Madhok et al. 1993; Maini et al. 2006; Pradhan et al. 2001; Ridker et al. 2021). In periodontitis, IL‐6 is generated locally in response to bacterial and inflammatory stimuli and subsequently enters the circulation, where persistent inflammation can amplify its levels through secondary production in extra‐oral tissues (D'Aiuto et al. 2004; Hajishengallis and Chavakis 2021; Kent et al. 1998; Lyngso et al. 2002; Mohamed‐Ali et al. 1997; Yamazaki et al. 1994). Moreover, several clinical studies and meta‐analyses have demonstrated that circulating IL‐6 levels decline following periodontal therapy (Ren and Li 2022), further supporting its role as a systemic marker of periodontal inflammation. Taken together, these properties justify the selection of circulating IL‐6 as a surrogate marker to evaluate the systemic impact of periodontitis.

Subgroup and meta‐regression analyses revealed that experimental periodontitis in mouse models induced by ligature placement produced significantly greater elevations of circulating IL‐6 than human periodontitis, indicating that systemic inflammation in ligature‐induced models is exaggerated. Ligature placement in mice provokes acute and intense local inflammation through mechanical tissue disruption and rapid bacterial overgrowth, leading to severe alveolar bone loss within 1–2 weeks (de Molon et al. 2014; Marchesan et al. 2018). This sharply contrasts with the chronic and multifactorial progression of human periodontitis, which typically develops over months to years (Papapanou et al. 2018a). These differences drive a disproportionately strong local inflammatory response and greater systemic cytokine spillover (Abe and Hajishengallis 2013; Marchesan et al. 2018). Furthermore, experimental mice are typically young, systemically healthy, and maintained under specific‐pathogen‐free conditions, resulting in a relatively low basal inflammatory state compared to humans (Beura et al. 2016; Hamilton et al. 2020; Tao and Reese 2017). In contrast, increased microbial exposure has been shown to elevate baseline inflammatory activity (Beura et al. 2016; Huggins et al. 2019). Therefore, even under comparable microbial challenges, the lower baseline in mice may result in a larger relative increase in inflammatory markers, leading to a more pronounced difference compared to controls.

The exaggerated IL‐6 response observed in ligature‐induced mouse models may not accurately reflect systemic inflammatory patterns in humans. Importantly, circulating IL‐6 levels show dose‐dependent relationships with systemic disease outcomes in humans (Danesh et al. 2008; Ridker et al. 2018; X. Wang et al. 2013). Specifically, larger increases in IL‐6 are associated with greater risk or severity of systemic diseases. In large prospective cohorts, a two–standard deviation increase in circulating IL‐6 was associated with a 2.14‐fold higher risk of coronary heart disease (Danesh et al. 2008). In the CANTOS trial, greater reductions in on‐treatment IL‐6 were linked to proportionally fewer cardiovascular events (Ridker et al. 2018). Likewise, each one–log pg/mL increase in circulating IL‐6 was associated with a 1.31‐fold higher risk of developing type 2 diabetes (Wang et al. 2013). While these associations arise from multifactorial human conditions and, in the case of CANTOS, from populations with established cardiovascular disease, they nonetheless highlight the biological relevance of IL‐6 magnitude. In this context, the markedly elevated IL‐6 responses observed in ligature‐induced models may overestimate the magnitude of systemic inflammation relative to human periodontitis, warranting caution when interpreting their translational relevance.

In contrast to ligature‐induced models, elevations of IL‐6 levels in oral inoculation models did not significantly differ from those observed in human periodontitis, suggesting that systemic inflammation in this model may be more comparable to the human condition. This relatively modest systemic response likely reflects the gradual progression of alveolar bone loss, with significant changes typically emerging after 4–8 weeks of repeated bacterial challenge (Nishimura et al. 2025). Oral inoculation thereby better mimics the chronic and natural course of periodontal pathology by allowing interactions with the resident oral microbiota. Nevertheless, several limitations remain. Oral inoculation models usually require antibiotic pretreatment to facilitate colonization, which perturbs the native microbiota and limits the establishment of stable, polymicrobial biofilms (Liu and Choi 2022; Papadopoulos et al. 2014; Wang et al. 2009). Moreover, the microbial communities that develop after repeated inoculation—often with single or limited bacterial strains—lack the diversity and ecological complexity of the human oral microbiome, which comprises more than 700 species and functions through polymicrobial synergy (Hajishengallis et al. 2023). Consequently, host–microbe interactions in this model do not fully recapitulate the chronic and dynamic interplay between pathogens, commensals, and the host immune system that characterizes human disease. Taken together, these considerations underscore the need to develop experimental models that more closely mimic human periodontitis to accurately assess its systemic impact and elucidate its underlying biological mechanisms.

Despite these insights, several limitations should be acknowledged. First, the number of available animal studies—particularly inoculation models—was limited, which may have reduced statistical power. Second, substantial heterogeneity remained even after subgroup and meta‐regression analyses, likely reflecting both methodological and biological differences across studies. In human studies, heterogeneity was particularly pronounced (*I* ^2^ = 99.1%), likely reflecting variability in study design (e.g., case–control versus cross‐sectional), differences in participant characteristics such as body mass index and metabolic status, and inconsistencies in sample collection protocols, including fasting versus non‐fasting conditions. These factors may have influenced baseline IL‐6 levels and contributed to variability in effect estimates across studies. In contrast, heterogeneity in animal studies, although still considerable (*I* ^2^ = 88.1%), appeared to be primarily driven by experimental factors, particularly differences in the duration of ligature‐induced periodontitis, which directly affect the magnitude of the inflammatory response. Third, most analyses were based on study‐level data, precluding adjustment for individual‐level confounders. Finally, although IL‐6 is a well‐established pro‐inflammatory cytokine, it represents only one facet of systemic immunity and should be interpreted alongside other biomarkers.

## 5 Conclusions

This systematic review and meta‐analysis demonstrated a consistent association between periodontitis and elevated circulating IL‐6 levels in both humans and mice. However, the magnitude of this effect was substantially greater in mouse models—particularly those using ligature‐induced periodontitis—indicating that systemic inflammatory responses in preclinical studies may exceed those typically observed in clinical settings. These findings highlight the need for cautious interpretation when extrapolating animal data to humans and emphasize that, although mouse models provide valuable mechanistic insights, their translational relevance is limited by differences in disease induction and host characteristics. Future research should focus on developing experimental models that more faithfully replicate the chronic, polymicrobial, and multifactorial nature of human periodontitis to better elucidate its systemic consequences. In addition, given the dynamic nature of inflammatory biomarkers in periodontitis, longitudinal assessment of circulating markers such as IL‐6 may further improve understanding of the temporal relationship between oral inflammation and systemic responses.

## Author Contributions

**Kyeongho Lee:** data curation, validation. **Jeong‐Min Lee:** writing – original draft, data curation, formal analysis, visualization. **June Yeon Kim:** data curation, validation. **Sung‐Jin Kim:** conceptualization, supervision, methodology, funding acquisition, writing – review and editing, project administration, resources. **Hun Jin Jeong:** data curation, validation.

## Funding

This work was supported by the National Research Foundation of Korea (NRF) grant funded by the Korea government (MSIT) (RS‐2023‐00210013 to S.‐J.K.). This research was also partially supported by a Research Grant funded by the School of Dentistry, Seoul National University (860‐20230089).

## Conflicts of Interest

The authors declare no conflicts of interest.

## Supporting information


**Data S1:** odi70367‐sup‐0001‐supinfo01.docx.

**Table S1:** Studies excluded after full text evaluation and reasons for exclusion.
**Table S2:** Characteristics of human studies included in the systematic review and meta‐analysis of circulating IL‐6 levels.
**Table S3:** Characteristics of mouse studies included in the systematic review and meta‐analysis of circulating IL‐6 levels.

**Figure S1:** Detailed risk of bias assessment for individual human studies evaluated with the ROBINS‐E tool.
**Figure S2:** Detailed risk of bias assessment for individual mouse studies evaluated with the SYRCLE risk of bias tool.
**Figure S3:** Leave‐one‐out sensitivity analyses of circulating IL‐6 levels in periodontitis compared with controls. (A) Human studies. (B) Mouse studies. Each row represents the pooled effect size after omitting the corresponding study. Effect sizes are shown as standardized mean differences (SMD) with 95% confidence intervals (CI), estimated using random‐effects models.
**Figure S4:** Funnel plots assessing publication bias in studies of circulating IL‐6 levels in periodontitis compared with controls. (A) Human studies. (B) Inoculation‐induced mouse models. (C) All mouse studies. (D) Ligature‐induced mouse models. Each circle represents an individual study plotted by its standardized mean difference (SMD) against the standard error.

## Data Availability

The data that support the findings of this study are available from the corresponding author upon reasonable request.
