# Supplementary material for: Comparative Circulating Interleukin‐6 Levels in Human and Mouse Periodontitis: A Systematic Review and Meta‐Analysis
Source: Oral Dis. 2026 May 20;32(9):2660–71. doi: 10.1111/odi.70367 (PMC13752615; doi:10.1111/odi.70367)
Supplement: Supplementary file 1 [file ODI-32-2660-s001.pdf]

Figure S1

Risk of bias domains

Study	D1	D2	D3	D4	D5	D6	D7	Overall
Ali et al. (2023)	-	-	+	+	+	+	+	-
Bretz et al. (2005)	+	+	-	+	-	+	-	-
Fredriksson et al. (2002)	-	+	-	+	-	+	-	-
George & Janam (2013)	-	+	-	+	-	+	-	-
Gümüş et al. (2014)	-	+	-	+	+	+	-	-
Hetta et al. (2020)	-	+	-	+	+	+	-	-
Keles et al. (2014)	-	+	-	+	+	+	-	-
Kurgan et al. (2022)	-	-	+	+	+	+	+	-
Lappin et al. (2011)	-	+	-	+	+	+	-	-
Lappin et al. (2015)	-	+	-	+	+	+	-	-
Leite et al. (2019)	-	+	-	+	+	+	-	-
Lobao et al. (2019)	X	-	+	-	-	-	-	X
Loo et al. (2012)	-	+	-	+	+	+	-	-
Marcaccini et al. (2009)	-	+	-	+	-	+	-	-
Miranda et al. (2019)	-	+	-	+	+	+	-	-
Miyazawa et al. (2012)	-	+	-	+	+	+	-	-
Nakajima et al. (2010)	-	+	-	+	+	+	-	-
Önder et al. (2023)	-	-	+	+	+	+	+	-
Rabelo et al. (2021)	-	-	+	-	+	+	+	-
Şengül et al. (2022)	-	-	+	+	+	+	+	-
Tian et al. (2013)	X	+	X	+	+	+	-	X
Turkmen et al. (2023)	X	+	X	+	+	+	-	X
Wohlfeil et al. (2012)	X	+	X	+	+	+	-	X
Yamazaki et al. (2005)	-	+	-	+	+	+	-	-
Yilmaz et al. (2022)	-	-	+	+	+	+	+	-
Zimmermann et al. (2013)	-	-	+	+	+	+	+	-

Domains:
D1: Bias due to confounding.
D2: Bias arising from measurement of the exposure.
D3: Bias in selection of participants into the study (or into the analysis).
D4: Bias due to post-exposure interventions.
D5: Bias due to missing data.
D6: Bias arising from measurement of the outcome.
D7: Bias in selection of the reported result.

Judgement
X High
- Some concerns
+ Low

Figure S2

Risk of bias domains

Study	D1	D2	D3	D4	D5	D6	D7	D8	D9	D10	Overall
Furutama et al. (2020)	-	+	-	-	-	-	+	+	+	+	-
Han et al. (2021)	-	+	-	-	-	-	+	+	+	+	-
Han et al. (2023)	-	+	-	-	-	-	+	+	+	+	-
Kang et al. (2022)	-	+	-	-	-	-	+	+	+	+	-
Kim et al. (2023)	-	+	-	-	-	-	+	+	+	+	-
Li et al. (2021)	+	+	-	-	-	-	+	+	+	+	-
Marruganti et al. (2024)	-	+	-	-	-	-	+	+	+	+	-
Matsuda et al. (2016)	-	+	-	-	-	-	+	+	+	+	-
Queiroz et al. (2012)	-	+	-	-	-	-	+	+	+	+	-
Tamura et al. (2022)	-	+	-	-	-	-	+	+	+	+	-
Xue et al. (2020)	-	+	-	-	-	-	+	+	+	+	-
Yang et al. (2025)	-	+	-	-	-	-	+	+	+	+	-

D1: Sequence generation
D2: Baseline characteristics
D3: Allocation concealment
D4: Random housing
D5: Blinding of caregivers
D6: Random outcome assessment
D7: Blinding of outcome assessment
D8: Incomplete outcome data
D9: Selective outcome reporting
D10: Other bias

Judgement
- Unclear
+ Low

Figure S3

A

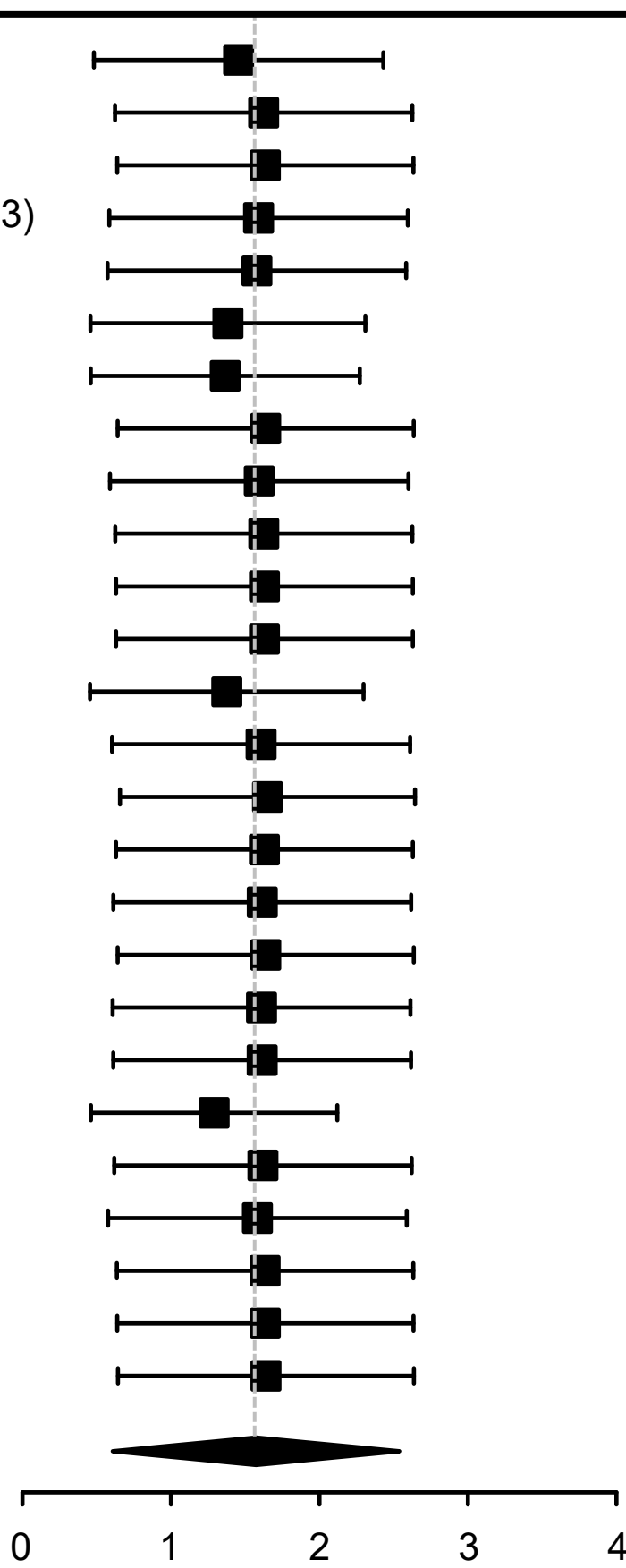

Study	SMD	95%-CI	P-value	Tau2	Tau	I2
Omitting Ali et al. (2023)	1.46	[0.48, 2.43]	< 0.01	6.0559	2.4609	99.1%
Omitting Bretz et al. (2005)	1.62	[0.62, 2.63]	< 0.01	6.3885	2.5275	98.8%
Omitting Fredriksson et al. (2002)	1.64	[0.64, 2.63]	< 0.01	6.3467	2.5193	99.2%
Omitting George & Janam et al. (2013)	1.59	[0.58, 2.60]	< 0.01	6.4463	2.5390	99.2%
Omitting Gümüş et al. (2014)	1.58	[0.57, 2.58]	< 0.01	6.4511	2.5399	99.2%
Omitting Hetta et al. (2020)	1.38	[0.46, 2.31]	< 0.01	5.4557	2.3357	99.0%
Omitting Keles et al. (2014)	1.37	[0.46, 2.27]	< 0.01	5.2445	2.2901	99.0%
Omitting Kurgan et al. (2022)	1.64	[0.64, 2.64]	< 0.01	6.3398	2.5179	99.2%
Omitting Lappin et al. (2011)	1.59	[0.59, 2.60]	< 0.01	6.4439	2.5385	99.2%
Omitting Lappin et al. (2015)	1.63	[0.62, 2.63]	< 0.01	6.3820	2.5263	99.2%
Omitting Leite et al. (2019)	1.63	[0.63, 2.63]	< 0.01	6.3700	2.5239	99.1%
Omitting Lobao et al. (2019)	1.38	[0.63, 2.63]	< 0.01	6.3698	2.5239	99.1%
Omitting Loo et al. (2012)	1.38	[0.45, 2.30]	< 0.01	5.3901	2.3216	98.9%
Omitting Marcaccini et al. (2009)	1.61	[0.60, 2.61]	< 0.01	6.4242	2.5346	99.2%
Omitting Miranda et al. (2019)	1.65	[0.66, 2.64]	< 0.01	6.2927	2.5085	99.1%
Omitting Miyazawa et al. (2012)	1.63	[0.63, 2.63]	< 0.01	6.3708	2.5240	99.1%
Omitting Nakajima et al. (2010)	1.61	[0.61, 2.62]	< 0.01	6.4109	2.5320	99.1%
Omitting Önder et al. (2023)	1.64	[0.64, 2.64]	< 0.01	6.3401	2.5180	99.1%
Omitting Rabelo et al. (2021)	1.61	[0.61, 2.61]	< 0.01	6.4168	2.5331	99.2%
Omitting Şengül et al. (2022)	1.61	[0.61, 2.62]	< 0.01	6.4101	2.5318	99.2%
Omitting Tian et al. (2013)	1.29	[0.46, 2.12]	< 0.01	4.3513	2.0860	98.7%
Omitting Turkmen et al. (2023)	1.62	[0.62, 2.62]	< 0.01	6.3969	2.5292	99.2%
Omitting Wohlfeil et al. (2012)	1.58	[0.58, 2.59]	< 0.01	6.4520	2.5401	99.2%
Omitting Yamazaki et al. (2005)	1.63	[0.64, 2.63]	< 0.01	6.3549	2.5209	99.2%
Omitting Yilmaz et al. (2022)	1.63	[0.64, 2.63]	< 0.01	6.3478	2.5195	99.2%
Omitting Zimmermann et al. (2013)	1.64	[0.64, 2.63]	< 0.01	6.3339	2.5167	99.2%
**Random effects model**	**1.57**	**[0.61,2.54]**	**< 0.01**	**6.1771**	**2.4854**	**99.1%**

B

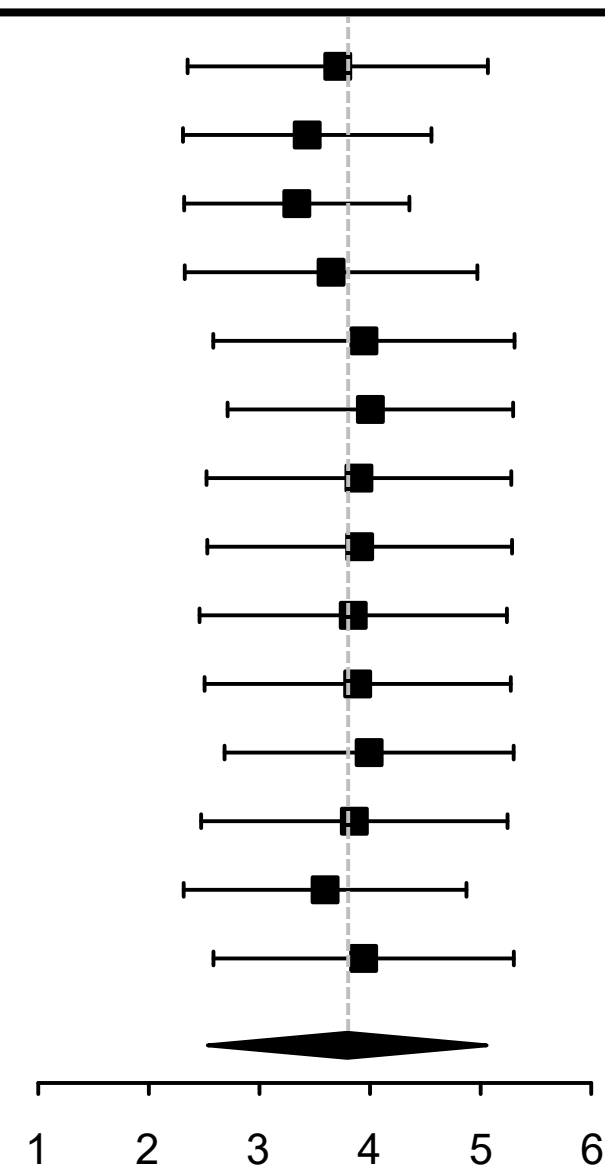

Study	SMD	95%-CI	P-value	Tau2	Tau	I2
Omitting Furutama et al. (2020)a	3.72	[2.36, 5.08]	< 0.01	5.3101	2.3044	89.1%
Omitting Furutama et al. (2020)b	3.44	[2.32, 4.57]	< 0.01	3.4781	1.8650	84.9%
Omitting Furutama et al. (2020)c	3.35	[2.33, 4.37]	< 0.01	2.7642	1.6626	81.8%
Omitting Han et al. (2021)	3.66	[2.34, 4.98]	< 0.01	5.0150	2.2394	88.7%
Omitting Han et al. (2023)	3.96	[2.60, 5.32]	< 0.01	5.3066	2.3036	88.2%
Omitting Kang et al. (2022)	4.02	[2.72, 5.31]	< 0.01	4.6859	2.1647	87.1%
Omitting Kim et al. (2023)	3.91	[2.53, 5.29]	< 0.01	5.4802	2.3410	89.3%
Omitting Li et al. (2021)	3.92	[2.54, 5.30]	< 0.01	5.4695	2.3387	89.1%
Omitting Marruganti et al. (2024)	3.86	[2.47, 5.25]	< 0.01	5.5869	2.3637	89.4%
Omitting Matsuda et al. (2016)	3.90	[2.52, 5.29]	< 0.01	5.5310	2.3518	89.2%
Omitting Queiroz et al. (2012)	4.00	[2.70, 5.31]	< 0.01	4.8343	2.1987	87.7%
Omitting Tamura et al. (2022)	3.87	[2.49, 5.26]	< 0.01	5.5589	2.3577	89.5%
Omitting Xue et al. (2020)	3.61	[2.33, 4.88]	< 0.01	4.6634	2.1595	88.1%
Omitting Yang et al. (2025)	3.95	[2.60, 5.31]	< 0.01	5.2908	2.3002	88.7%
**Random effects model**	**3.79**	**[2.53, 5.05]**	**< 0.01**	**4.8793**	**2.2089**	**88.1%**

Figure S4

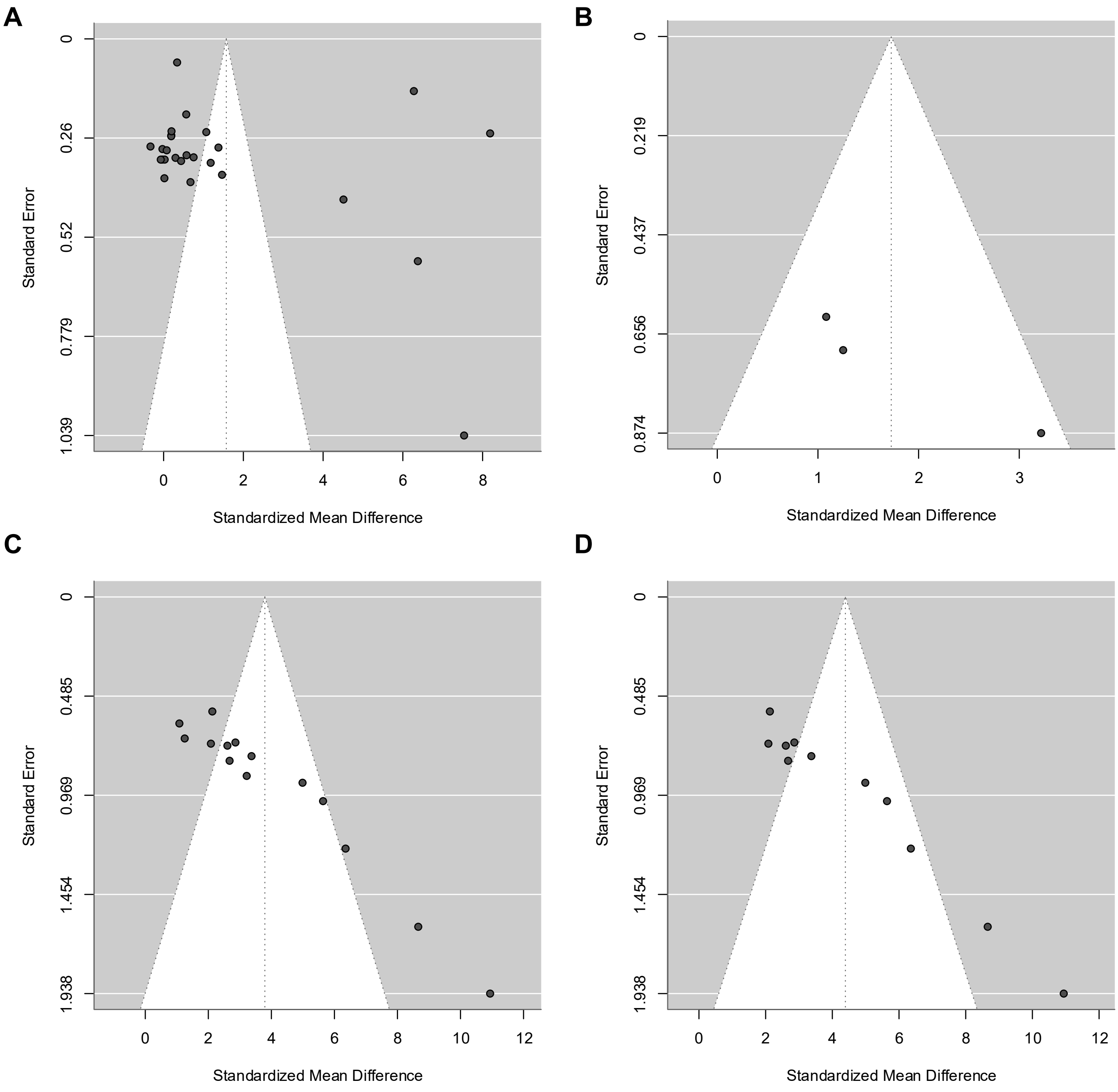

A
Standard Error
0
0.26
0.52
0.779
1.039
0
2
4
6
8
Standardized Mean Difference
B
Standard Error
0
0.219
0.437
0.656
0.874
0
1
2
3
Standardized Mean Difference
C
Standard Error
0
0.485
0.969
1.454
1.938
0
2
4
6
8
10
12
Standardized Mean Difference
D
Standard Error
0
0.485
0.969
1.454
1.938
0
2
4
6
8
10
12
Standardized Mean Difference
