# Supplementary material for: Comparative Circulating Interleukin‐6 Levels in Human and Mouse Periodontitis: A Systematic Review and Meta‐Analysis
Source: Oral Dis. 2026 May 20;32(9):2660–71. doi: 10.1111/odi.70367 (PMC13752615; doi:10.1111/odi.70367)
Supplement: Supplementary file 2 [file ODI-32-2660-s002.docx]

**Table S1.** Studies excluded after full text evaluation and reasons for exclusion

| **Reason for Exclusion** | **Publication** |
| --- | --- |
| Unable to estimate values | (Gani, Lakshmi, Krishnan, & Emmadi, 2009; Gani et al., 2012; Kaiser et al., 2018; Latorre Uriza et al., 2024; Loos, Craandijk, Hoek, Wertheim-van Dillen, & van der Velden, 2000; Medara et al., 2020; Suh, Kim, Lee, Kim, & Park, 2022; Yu et al., 2016; Yu et al., 2019; X. Zhang et al., 2013) |
| Unable to classify periodontitis stage | (Bahrani-Mougeot et al., 2008; D'Aiuto et al., 2010; D. H. Han, Shin, Kim, Paek, & Kim, 2012; Higashi et al., 2008; Kim, Sukhbaatar, Shin, Ahn, & Yoo, 2014; Masi et al., 2011; Mengel, Bacher, & Flores-De-Jacoby, 2002; Scherbaum et al., 2020) |
| Implausible or inconsistent data | (Becerik, Ozturk, Atmaca, Atilla, & Emingil, 2012; Buhlin, Gustafsson, Pockley, Frostegard, & Klinge, 2003; Kurgan et al., 2016; Murakami-Malaquias-da-Silva et al., 2020; Pirim Gorgun, Toker, Korkmaz, & Poyraz, 2017; Saadi-Thiers et al., 2013; Temelli et al., 2018; Toker, Gorgun, Korkmaz, Yuce, & Poyraz, 2018) |
| Aggressive or acute periodontitis only | (Davies et al., 2011; Haba et al., 2011; Y. Han, Huang, Meng, Zhan, & Hou, 2021; Keles Yucel & Balli, 2021; Robati, Ranjbari, Ghafourian Boroujerdnia, & Chinipardaz, 2011; Shi et al., 2015; Sun et al., 2009) |
| No healthy control group | (Furugen et al., 2008; Plachokova, Andreu-Sanchez, Noz, Fu, & Riksen, 2021; Rafique et al., 2021) |
| Included Stage I-II periodontitis | (Acharya, Thakur, Muddapur, & Kulkarni, 2016, 2018; Arvikar et al., 2021; Bakshi et al., 2018; Chang, Blanchard, Windsor, Gregory, & Hamada, 2020; Chen et al., 2008; Cicmil et al., 2023; Ding, Luo, Wen, Li, & Bao, 2022; Dopico et al., 2023; Duzagac et al., 2016; Eldzharov et al., 2021; Fuchter et al., 2025; Gangadhar, Ramesh, & Thomas, 2011; Y. Han et al., 2023; Ishida et al., 2012; Jiang, Zhou, Zhang, & Wen, 2021; Kajihara et al., 2022; Kobayashi, Ishida, & Yoshie, 2016; Leira et al., 2019; Leira et al., 2020; Li, He, Chen, & Luan, 2020; Longo et al., 2014; Lu et al., 2022; Lyra et al., 2024; Martinez-Herrera et al., 2018; Miyashita et al., 2012; Monteiro et al., 2009; Noz et al., 2021; Sert, Kirzioglu, Fentoglu, Aylak, & Mungan, 2011; Shimada et al., 2010; Storjord et al., 2022; Tabeta et al., 2018; Takahashi et al., 1994; Wang, Tang, & Xiao, 2023; Zekeridou, Mombelli, Cancela, Courvoisier, & Giannopoulou, 2019; J. Zhang et al., 2017) |
| Inappropriate periodontitis induction method | (Sato, Yokoji, Yamada, Nakajima, & Yamazaki, 2018; Scanu et al., 2019; Yuan, Gupte, Zelkha, & Amar, 2011) |

- Unable to estimate values = No quantitative IL-6 data reported, insufficient information to calculate mean and standard deviation, or unclear/incomplete reporting of sample size.
- Unable to classify periodontitis = Clinical information insufficient to determine or retrospectively classify periodontitis stage based on the 2018 AAP/EFP classification
- Implausible or inconsistent data = IL-6 data presented with implausible values, inconsistent units (e.g., ng/mL vs. pg/mL), or discrepancies across tables, figures, or text.
- Aggressive or acute periodontitis only = Studies exclusively including participants with aggressive or acute periodontitis only
- No healthy control group = Study lacked a periodontally healthy comparison group necessary for effect size estimation.
- Included stage I-II periodontitis = Studies included patients with Stage I or Stage II periodontitis.
- Inappropriate periodontitis induction method = Mouse studies were excluded if they did not induce periodontitis using ligature placement or oral bacterial inoculation, or if they failed to demonstrate resulting periodontal inflammation or bone destruction.

**REFERENCES (EXCLUDED STUDIES)**

Acharya, A. B., Thakur, S., Muddapur, M. V., & Kulkarni, R. D. (2016). Tumor necrosis factor-alpha, interleukin-4 and -6 in the serum of health, chronic periodontitis, and type 2 diabetes mellitus. *J Indian Soc Periodontol, 20*(5), 509–513. doi:10.4103/0972-124X.201694

Acharya, A. B., Thakur, S., Muddapur, M. V., & Kulkarni, R. D. (2018). Systemic Cytokines in Type 2 Diabetes Mellitus and Chronic Periodontitis. *Curr Diabetes Rev, 14*(2), 182–188. doi:10.2174/1573399812666161220144011

Arvikar, S. L., Hasturk, H., Strle, K., Stephens, D., Bolster, M. B., Collier, D. S., . . . Steere, A. C. (2021). Periodontal inflammation and distinct inflammatory profiles in saliva and gingival crevicular fluid compared with serum and joints in rheumatoid arthritis patients. *J Periodontol, 92*(10), 1379–1391. doi:10.1002/JPER.20-0051

Bahrani-Mougeot, F. K., Thornhill, M., Sasser, H., Marriott, I., Brennan, M. T., Papagerakis, S., . . . Lockhart, P. B. (2008). Systemic host immuno-inflammatory response to dental extractions and periodontitis. *Oral Surg Oral Med Oral Pathol Oral Radiol Endod, 106*(4), 534–541. doi:10.1016/j.tripleo.2008.02.011

Bakshi, D., Kaur, G., Singh, D., Sahota, J., Thakur, A., & Grover, S. (2018). Estimation of Plasma Levels of Tumor Necrosis Factor-a, Interleukin-4 and 6 in Patients with Chronic Periodontitis and Type II Diabetes Mellitus. *J Contemp Dent Pract, 19*(2), 166–169. doi:10.5005/jp-journals-10024-2231

Becerik, S., Ozturk, V. O., Atmaca, H., Atilla, G., & Emingil, G. (2012). Gingival crevicular fluid and plasma acute-phase cytokine levels in different periodontal diseases. *J Periodontol, 83*(10), 1304–1313. doi:10.1902/jop.2012.110616

Buhlin, K., Gustafsson, A., Pockley, A. G., Frostegard, J., & Klinge, B. (2003). Risk factors for cardiovascular disease in patients with periodontitis. *Eur Heart J, 24*(23), 2099–2107. doi:10.1016/j.ehj.2003.09.016

Chang, J., Blanchard, S. B., Windsor, L. J., Gregory, R. L., & Hamada, Y. (2020). Levels of growth factors from platelet-rich fibrin from chronic periodontitis versus periodontally healthy subjects: a pilot study. *Clin Oral Investig, 24*(2), 823–832. doi:10.1007/s00784-019-02944-7

Chen, Y. W., Umeda, M., Nagasawa, T., Takeuchi, Y., Huang, Y., Inoue, Y., . . . Ishikawa, I. (2008). Periodontitis may increase the risk of peripheral arterial disease. *Eur J Vasc Endovasc Surg, 35*(2), 153–158. doi:10.1016/j.ejvs.2007.08.016

Cicmil, S., Cicmil, A., Pavlic, V., Krunic, J., Sladoje Puhalo, D., Bokonjic, D., & Colic, M. (2023). Periodontal Disease in Young Adults as a Risk Factor for Subclinical Atherosclerosis: A Clinical, Biochemical and Immunological Study. *J Clin Med, 12*(6). doi:10.3390/jcm12062197

D'Aiuto, F., Nibali, L., Parkar, M., Patel, K., Suvan, J., & Donos, N. (2010). Oxidative stress, systemic inflammation, and severe periodontitis. *J Dent Res, 89*(11), 1241–1246. doi:10.1177/0022034510375830

Davies, R. C., Jaedicke, K. M., Barksby, H. E., Jitprasertwong, P., Al-Shahwani, R. M., Taylor, J. J., & Preshaw, P. M. (2011). Do patients with aggressive periodontitis have evidence of diabetes? A pilot study. *J Periodontal Res, 46*(6), 663–672. doi:10.1111/j.1600-0765.2011.01388.x

Ding, N., Luo, M., Wen, Y. H., Li, R. Y., & Bao, Q. Y. (2022). The Effects of Non-Surgical Periodontitis Therapy on the Clinical Features and Serological Parameters of Patients Suffering from Rheumatoid Arthritis as Well as Chronic Periodontitis. *J Inflamm Res, 15*, 177–185. doi:10.2147/JIR.S326896

Dopico, J., Botelho, J., Ouro, A., Dominguez, C., Machado, V., Aramburu-Nunez, M., . . . Leira, Y. (2023). Association between periodontitis and peripheral markers of innate immunity activation and inflammation. *J Periodontol, 94*(1), 11–19. doi:10.1002/JPER.22-0216

Duzagac, E., Cifcibasi, E., Erdem, M. G., Karabey, V., Kasali, K., Badur, S., & Cintan, S. (2016). Is obesity associated with healing after non-surgical periodontal therapy? A local vs. systemic evaluation. *J Periodontal Res, 51*(5), 604–612. doi:10.1111/jre.12340

Eldzharov, A., Kabaloeva, D., Nemeryuk, D., Goncharenko, A., Gatsalova, A., Ivanova, E., . . . Bourgeois, D. (2021). Evaluation of Microcirculation, Cytokine Profile, and Local Antioxidant Protection Indices in Periodontal Health, and Stage II, Stage III Periodontitis. *J Clin Med, 10*(6). doi:10.3390/jcm10061262

Fuchter, S. C. C., Stolarski, B., Manica, D., Chielle, E. O., de Resende, E. S. D. T., & Franco Vieira de Oliveira Maciel, S. (2025). Patients with periodontal disease demonstrates changes in purinergic and inflammatory markers in PBMCs, serum and saliva. *Purinergic Signal*. doi:10.1007/s11302-025-10082-x

Furugen, R., Hayashida, H., Yamaguchi, N., Yoshihara, A., Ogawa, H., Miyazaki, H., & Saito, T. (2008). The relationship between periodontal condition and serum levels of resistin and adiponectin in elderly Japanese. *J Periodontal Res, 43*(5), 556–562. doi:10.1111/j.1600-0765.2008.01085.x

Gangadhar, V., Ramesh, A., & Thomas, B. (2011). Correlation between leptin and the health of the gingiva: a predictor of medical risk. *Indian J Dent Res, 22*(4), 537–541. doi:10.4103/0970-9290.90292

Gani, D. K., Lakshmi, D., Krishnan, R., & Emmadi, P. (2009). Evaluation of C-reactive protein and interleukin-6 in the peripheral blood of patients with chronic periodontitis. *J Indian Soc Periodontol, 13*(2), 69–74. doi:10.4103/0972-124X.55840

Gani, D. K., Mallineni, S. K., Ambalavanan, Ramakrishnan, Deepalakshmi, & Emmadi, P. (2012). Estimation of the levels of C-reactive protein, interleukin-6, total leukocyte count, and differential count in peripheral blood smear of patients with chronic periodontitis in a South Indian population. *West Indian Med J, 61*(8), 826–831.

Haba, D., Teslaru, S., Ungureanu, D., Hodorog, D., Alecu, C., Benghiac, A. G., . . . Iordache, C. (2011). Evaluation of serum and gingival crevicular fluid C-reactive protein and IL-6 levels in patients with periodontitis and transient ischemic attacks. *Rom J Morphol Embryol, 52*(4), 1243–1247.

Han, D. H., Shin, H. S., Kim, M. S., Paek, D., & Kim, H. D. (2012). Group of serum inflammatory markers and periodontitis-metabolic syndrome coexistence in Koreans. *J Periodontol, 83*(5), 612–620. doi:10.1902/jop.2011.110304

Han, Y., Huang, W., Meng, H., Zhan, Y., & Hou, J. (2021). Pro-inflammatory cytokine interleukin-6-induced hepcidin, a key mediator of periodontitis-related anemia of inflammation. *J Periodontal Res, 56*(4), 690–701. doi:10.1111/jre.12865

Han, Y., Luo, Z., Yue, Z. G., Miao, L. L., Xv, M., Chang, S., . . . Hou, J. (2023). The tendency of anemia of inflammation in periodontal diseases. *Clin Sci (Lond), 137*(3), 251–264. doi:10.1042/CS20220524

Higashi, Y., Goto, C., Jitsuiki, D., Umemura, T., Nishioka, K., Hidaka, T., . . . Taguchi, A. (2008). Periodontal infection is associated with endothelial dysfunction in healthy subjects and hypertensive patients. *Hypertension, 51*(2), 446–453. doi:10.1161/HYPERTENSIONAHA.107.101535

Ishida, K., Kobayashi, T., Ito, S., Komatsu, Y., Yokoyama, T., Okada, M., . . . Yoshie, H. (2012). Interleukin-6 gene promoter methylation in rheumatoid arthritis and chronic periodontitis. *J Periodontol, 83*(7), 917–925. doi:10.1902/jop.2011.110356

Jiang, F., Zhou, Y., Zhang, R., & Wen, Y. (2021). miR-205 and HMGB1 expressions in chronic periodontitis patients and their associations with the inflammatory factors. *Am J Transl Res, 13*(8), 9224–9232.

Kaiser, F., Donos, N., Henderson, B., Alagarswamy, R., Pelekos, G., Boniface, D., & Nibali, L. (2018). Association between circulating levels of heat-shock protein 27 and aggressive periodontitis. *Cell Stress Chaperones, 23*(5), 847–856. doi:10.1007/s12192-018-0891-4

Kajihara, R., Sakai, H., Han, Y., Amari, K., Kawamoto, M., Hakoyama, Y., . . . Kurita, H. (2022). Presence of periodontitis may synergistically contribute to cancer progression via Treg and IL-6. *Sci Rep, 12*(1), 11584. doi:10.1038/s41598-022-15690-w

Keles Yucel, Z. P., & Balli, U. (2021). Leucine-rich alpha-2 glycoprotein (LRG): A novel acute phase protein expressed in Stage 3 Grade C periodontitis before and after periodontal therapy. *J Periodontol, 92*(1), 104–112. doi:10.1002/JPER.20-0358

Kim, H. D., Sukhbaatar, M., Shin, M., Ahn, Y. B., & Yoo, W. S. (2014). Validation of periodontitis screening model using sociodemographic, systemic, and molecular information in a Korean population. *J Periodontol, 85*(12), 1676–1683. doi:10.1902/jop.2014.140061

Kobayashi, T., Ishida, K., & Yoshie, H. (2016). Increased expression of interleukin-6 (IL-6) gene transcript in relation to IL-6 promoter hypomethylation in gingival tissue from patients with chronic periodontitis. *Arch Oral Biol, 69*, 89–94. doi:10.1016/j.archoralbio.2016.05.018

Kurgan, S., Fentoglu, O., Onder, C., Serdar, M., Eser, F., Tatakis, D. N., & Gunhan, M. (2016). The effects of periodontal therapy on gingival crevicular fluid matrix metalloproteinase-8, interleukin-6 and prostaglandin E2 levels in patients with rheumatoid arthritis. *J Periodontal Res, 51*(5), 586–595. doi:10.1111/jre.12337

Latorre Uriza, C., Roa, N. S., Velosa-Porras, J., Villamil Poveda, J. C., Otero, L., Ruiz, A. J., & Escobar Arregoces, F. M. (2024). Relationship between Carotid Intima-Media Thickness, Periodontal Disease, and Systemic Inflammation Biomarkers in an Adult Population. *Biomedicines, 12*(7). doi:10.3390/biomedicines12071425

Leira, Y., Ameijeira, P., Dominguez, C., Lopez-Arias, E., Avila-Gomez, P., Perez-Mato, M., . . . Blanco, J. (2019). Periodontal inflammation is related to increased serum calcitonin gene-related peptide levels in patients with chronic migraine. *J Periodontol, 90*(10), 1088–1095. doi:10.1002/JPER.19-0051

Leira, Y., Carballo, A., Orlandi, M., Aldrey, J. M., Pias-Peleteiro, J. M., Moreno, F., . . . Blanco, J. (2020). Periodontitis and systemic markers of neurodegeneration: A case-control study. *J Clin Periodontol, 47*(5), 561–571. doi:10.1111/jcpe.13267

Li, P., He, L., Chen, Z. B., & Luan, Q. X. (2020). Biomarkers in Metabolic Syndrome Patients with Chronic Periodontitis. *Chin J Dent Res, 23*(3), 191–197. doi:10.3290/j.cjdr.a45223

Longo, P. L., Artese, H. P., Rabelo, M. S., Kawamoto, D., Foz, A. M., Romito, G. A., . . . Mayer, M. P. (2014). Serum levels of inflammatory markers in type 2 diabetes patients with chronic periodontitis. *J Appl Oral Sci, 22*(2), 103–108. doi:10.1590/1678-775720130540

Loos, B. G., Craandijk, J., Hoek, F. J., Wertheim-van Dillen, P. M., & van der Velden, U. (2000). Elevation of systemic markers related to cardiovascular diseases in the peripheral blood of periodontitis patients. *J Periodontol, 71*(10), 1528–1534. doi:10.1902/jop.2000.71.10.1528

Lu, H., Wu, H., Yang, Y., Feng, X., Ma, X., Xie, Y., . . . Ye, W. (2022). Relationship between chronic periodontitis and inflammatory cytokines in patients undergoing maintenance hemodialysis. *Clin Oral Investig, 26*(11), 6699–6709. doi:10.1007/s00784-022-04629-0

Lyra, P., Botelho, J., Rota, S., Poplawska-Domaszewicz, K., Machado, V., Guerreiro, D., . . . Chaudhuri, K. R. (2024). Non-Surgical Periodontal Therapy's Influence on Alpha-Synuclein and Inflammatory Marker Levels: A Pilot Study. *J Clin Med, 13*(12). doi:10.3390/jcm13123586

Martinez-Herrera, M., Silvestre, F. J., Silvestre-Rangil, J., Lopez-Domenech, S., Banuls, C., & Rocha, M. (2018). Levels of serum retinol-binding protein 4 before and after non-surgical periodontal treatment in lean and obese subjects: An interventional study. *J Clin Periodontol, 45*(3), 336–344. doi:10.1111/jcpe.12840

Masi, S., Salpea, K. D., Li, K., Parkar, M., Nibali, L., Donos, N., . . . Humphries, S. E. (2011). Oxidative stress, chronic inflammation, and telomere length in patients with periodontitis. *Free Radic Biol Med, 50*(6), 730–735. doi:10.1016/j.freeradbiomed.2010.12.031

Medara, N., Lenzo, J. C., Walsh, K. A., Darby, I. B., O'Brien-Simpson, N. M., & Reynolds, E. C. (2020). T helper 17 cell-related cytokines in serum and saliva during management of periodontitis. *Cytokine, 134*, 155186. doi:10.1016/j.cyto.2020.155186

Mengel, R., Bacher, M., & Flores-De-Jacoby, L. (2002). Interactions between stress, interleukin-1beta, interleukin-6 and cortisol in periodontally diseased patients. *J Clin Periodontol, 29*(11), 1012–1022. doi:10.1034/j.1600-051x.2002.291106.x

Miyashita, H., Honda, T., Maekawa, T., Takahashi, N., Aoki, Y., Nakajima, T., . . . Yamazaki, K. (2012). Relationship between serum antibody titres to Porphyromonas gingivalis and hs-CRP levels as inflammatory markers of periodontitis. *Arch Oral Biol, 57*(6), 820–829. doi:10.1016/j.archoralbio.2011.11.008

Monteiro, A. M., Jardini, M. A., Alves, S., Giampaoli, V., Aubin, E. C., Figueiredo Neto, A. M., & Gidlund, M. (2009). Cardiovascular disease parameters in periodontitis. *J Periodontol, 80*(3), 378–388. doi:10.1902/jop.2009.080431

Murakami-Malaquias-da-Silva, F., Rosa, E. P., Oliveira, J. G., Avelar, I. S., Palma-Cruz, M., Fernandes Silva, J. G., . . . Horliana, A. C. (2020). The role of periodontal treatment associated with photodynamic therapy on the modulation of systemic inflammation in the experimental model of asthma and periodontitis. *Photodiagnosis Photodyn Ther, 29*, 101619. doi:10.1016/j.pdpdt.2019.101619

Noz, M. P., Plachokova, A. S., Smeets, E. M. M., Aarntzen, E., Bekkering, S., Vart, P., . . . Riksen, N. P. (2021). An Explorative Study on Monocyte Reprogramming in the Context of Periodontitis In Vitro and In Vivo. *Front Immunol, 12*, 695227. doi:10.3389/fimmu.2021.695227

Pirim Gorgun, E., Toker, H., Korkmaz, E. M., & Poyraz, O. (2017). IL-6 and IL-10 gene polymorphisms in patients with aggressive periodontitis: effects on GCF, serum and clinic parameters. *Braz Oral Res, 31*, e12. doi:10.1590/1807-3107BOR-2017.vol31.0012

Plachokova, A. S., Andreu-Sanchez, S., Noz, M. P., Fu, J., & Riksen, N. P. (2021). Oral Microbiome in Relation to Periodontitis Severity and Systemic Inflammation. *Int J Mol Sci, 22*(11). doi:10.3390/ijms22115876

Rafique, S., Askari, S. N., Majeed, M. M., Khan, A. M., Kanwal, S., & Sattar, F. A. (2021). Association of Vitamin D Binding Protein with Interleukin-6 levels in patients with periodontitis. *Rawal Medical Journal, 46*(3), 620–624.

Robati, M., Ranjbari, A., Ghafourian Boroujerdnia, M., & Chinipardaz, Z. (2011). Detection of IL-4, IL-6 and IL-12 serum levels in generalized aggressive periodontitis. *Iran J Immunol, 8*(3), 170–175.

Saadi-Thiers, K., Huck, O., Simonis, P., Tilly, P., Fabre, J. E., Tenenbaum, H., & Davideau, J. L. (2013). Periodontal and systemic responses in various mice models of experimental periodontitis: respective roles of inflammation duration and Porphyromonas gingivalis infection. *J Periodontol, 84*(3), 396–406. doi:10.1902/jop.2012.110540

Sato, K., Yokoji, M., Yamada, M., Nakajima, T., & Yamazaki, K. (2018). An orally administered oral pathobiont and commensal have comparable and innocuous systemic effects in germ-free mice. *J Periodontal Res, 53*(6), 950–960. doi:10.1111/jre.12593

Scanu, A., Giraudo, C., Galuppini, F., Lazzarin, V., Pennelli, G., Sivolella, S., . . . Punzi, L. (2019). Periodontal Injection of Lipopolysaccharide Promotes Arthritis Development in Mice. *Inflammation, 42*(3), 1117–1128. doi:10.1007/s10753-019-00975-6

Scherbaum, I., Heidecke, H., Bunte, K., Peters, U., Beikler, T., & Boege, F. (2020). Autoantibodies against M(5)-muscarinic and beta(1)-adrenergic receptors in periodontitis patients. *Aging (Albany NY), 12*(16), 16609–16620. doi:10.18632/aging.103864

Sert, T., Kirzioglu, F. Y., Fentoglu, O., Aylak, F., & Mungan, T. (2011). Serum placental growth factor, vascular endothelial growth factor, soluble vascular endothelial growth factor receptor-1 and -2 levels in periodontal disease, and adverse pregnancy outcomes. *J Periodontol, 82*(12), 1735–1748. doi:10.1902/jop.2011.100740

Shi, D., Liu, Y. Y., Li, W., Zhang, X., Sun, X. J., Xu, L., . . . Meng, H. X. (2015). Association between plasma leptin level and systemic inflammatory markers in patients with aggressive periodontitis. *Chin Med J (Engl), 128*(4), 528–532. doi:10.4103/0366-6999.151110

Shimada, Y., Komatsu, Y., Ikezawa-Suzuki, I., Tai, H., Sugita, N., & Yoshie, H. (2010). The effect of periodontal treatment on serum leptin, interleukin-6, and C-reactive protein. *J Periodontol, 81*(8), 1118–1123. doi:10.1902/jop.2010.090741

Storjord, E., Airila-Mansson, S., Karlsen, K., Madsen, M., Dahl, J. A., Landsem, A., . . . Brekke, O. L. (2022). Dental and Periodontal Health in Acute Intermittent Porphyria. *Life (Basel), 12*(8). doi:10.3390/life12081270

Suh, J. S., Kim, S. Y. J., Lee, S. H., Kim, R. H., & Park, N. H. (2022). Hyperlipidemia is necessary for the initiation and progression of atherosclerosis by severe periodontitis in mice. *Mol Med Rep, 26*(2). doi:10.3892/mmr.2022.12789

Sun, X. J., Meng, H. X., Shi, D., Xu, L., Zhang, L., Chen, Z. B., . . . Ren, X. Y. (2009). Elevation of C-reactive protein and interleukin-6 in plasma of patients with aggressive periodontitis. *J Periodontal Res, 44*(3), 311–316. doi:10.1111/j.1600-0765.2008.01131.x

Tabeta, K., Hosojima, M., Nakajima, M., Miyauchi, S., Miyazawa, H., Takahashi, N., . . . Yoshie, H. (2018). Increased serum PCSK9, a potential biomarker to screen for periodontitis, and decreased total bilirubin associated with probing depth in a Japanese community survey. *J Periodontal Res, 53*(3), 446–456. doi:10.1111/jre.12533

Takahashi, K., Takashiba, S., Nagai, A., Takigawa, M., Myoukai, F., Kurihara, H., & Murayama, Y. (1994). Assessment of interleukin-6 in the pathogenesis of periodontal disease. *J Periodontol, 65*(2), 147–153. doi:10.1902/jop.1994.65.2.147

Temelli, B., Yetkin Ay, Z., Savas, H. B., Aksoy, F., Kumbul Doguc, D., Uskun, E., & Varol, E. (2018). Circulation levels of acute phase proteins pentraxin 3 and serum amyloid A in atherosclerosis have correlations with periodontal inflamed surface area. *J Appl Oral Sci, 26*, e20170322. doi:10.1590/1678-7757-2017-0322

Toker, H., Gorgun, E. P., Korkmaz, E. M., Yuce, H. B., & Poyraz, O. (2018). The effects of IL-10 gene polymorphism on serum, and gingival crevicular fluid levels of IL-6 and IL-10 in chronic periodontitis. *J Appl Oral Sci, 26*, e20170232. doi:10.1590/1678-7757-2017-0232

Wang, X., Tang, Y., & Xiao, R. (2023). Chemerin contributes to inflammatory responses and suppresses osteogenic differentiation in chronic periodontitis. *Oral Dis, 29*(4), 1706–1714. doi:10.1111/odi.14130

Yu, T., Zhao, L., Huang, X., Ma, C., Wang, Y., Zhang, J., & Xuan, D. (2016). Enhanced Activity of the Macrophage M1/M2 Phenotypes and Phenotypic Switch to M1 in Periodontal Infection. *J Periodontol, 87*(9), 1092–1102. doi:10.1902/jop.2016.160081

Yu, T., Zhao, L., Huang, X., Xie, B., Zhang, J., & Xuan, D. (2019). Aberrant Periodontal and Systemic Immune Response of Overweight Rodents to Periodontal Infection. *Biomed Res Int, 2019*, 9042542. doi:10.1155/2019/9042542

Yuan, H., Gupte, R., Zelkha, S., & Amar, S. (2011). Receptor activator of nuclear factor kappa B ligand antagonists inhibit tissue inflammation and bone loss in experimental periodontitis. *J Clin Periodontol, 38*(11), 1029–1036. doi:10.1111/j.1600-051X.2011.01780.x

Zekeridou, A., Mombelli, A., Cancela, J., Courvoisier, D., & Giannopoulou, C. (2019). Systemic inflammatory burden and local inflammation in periodontitis: What is the link between inflammatory biomarkers in serum and gingival crevicular fluid? *Clin Exp Dent Res, 5*(2), 128–135. doi:10.1002/cre2.162

Zhang, J., Zhang, A. M., Zhang, Z. M., Jia, J. L., Sui, X. X., Yu, L. R., & Liu, H. T. (2017). Efficacy of combined orthodontic-periodontic treatment for patients with periodontitis and its effect on inflammatory cytokines: A comparative study. *Am J Orthod Dentofacial Orthop, 152*(4), 494–500. doi:10.1016/j.ajodo.2017.01.028

Zhang, X., Meng, H., Sun, X., Xu, L., Zhang, L., Shi, D., . . . Chen, Z. (2013). Elevation of vitamin D-binding protein levels in the plasma of patients with generalized aggressive periodontitis. *J Periodontal Res, 48*(1), 74–79. doi:10.1111/j.1600-0765.2012.01505.x

**Table S2.** Characteristics of human studies included in the systematic review and meta-analysis of circulating IL-6 levels.

| Author(s)  Year | Country | Study design | Sample size | %Males | Males ratio (PD:C) | Mean age (year) | Age Ratio (PD:C) | Diagnostic Criteria for PD | Diagnostic thresholds (Secure or  Insecure) | Blood collection (serum or  Plasma) | Blood test  (fasting or  non-fasting) | BMI control |
| --- | --- | --- | --- | --- | --- | --- | --- | --- | --- | --- | --- | --- |
| Ali et al.  (2005) | Iraq | Case-Control | 80 | 50 | 1 | 33.0 | 1.11 | CAL = 5.45(0.90)mm | Secure | Serum | NI | NO |
| Bretz et al.  (2005) | United States | Cross-sectional | 1131 | 50 | 1 | 72.7 | 1 | probing depth of 6 mm or more | Secure | Plasma | NI | Yes |
| Fredriksson et al.  (2002) | Sweden | Case-Control | 30 | 40 | 1 | 48.93 | 1.07 | ≥6 sites with CAL >5 mm | Secure | Plasma | NI | NI |
| George &  Janam  (2013) | India | Case-Control | 45 | 53.3 | 0.8 | 42.38 | 0.97 | CAL ≥5 mm in ≥50% of teeth | Secure | Serum | Fasting | Yes |
| Gümüş  et al.  (2014) | Turkey | Case-Control | 4c0 | 60 | 1 | 43.8 | 1.39 | ≥4 teeth in each jaw with PD ≥5 mm, CAL ≥4 mm, and ≥50% alveolar bone loss in ≥2 quadrants, with BOP at >80% of sites | Secure | Serum | Fasting | NI |
| Hetta et al.  (2020) | Egypt | Case-Control | 75 | 60 | 0.9 | 36.98 | 1.05 | PPD ≥ 6 mm, CAL ≥ 5 mm, bone loss in ≥6 teeth | Secure | Serum | NI | NI |
| Keles et al.  (2014) | Turkey | Cross-sectional | 30 | 50 | 1.14 | 40.34 | 1.12 | GI ≥ 2, PD ≥ 4 mm with BOP, CAL ≥ 5 mm, bone loss | Secure | Serum | NI | NI |
| Kurgan  et al.  (2022) | Turkey | Cross-sectional | 40 | 47.5 | 1.38 | 40.6 | 1.05 | Stage III Grade B generalized periodontitis based on 2018 AAP/EFP classification | Secure | Serum | NI | NI |
| Lappin  et al.  (2011) | UK | Case-Control | 113 | 43.4 | 0.88 | 44.33 | 1.38 | ≥2 pockets >5 mm, CAL ≥5 mm, ≥16 teeth including ≥4 molars in different quadrants | Secure | Plasma | NI | NI |
| Lappin  et al.  (2015) | UK | Cross-sectional | 42 | 50 | 1.11 | 32.7 | 1.03 | ≥2 sites with PD and CAL ≥5 mm; full-mouth 6-point PD, CAL, and BOP charting conducted | Secure | Plasma | NI | NI |
| Leite et al.  (2019) | Brazil | Case-Control | 63 | 39.7 | 0.99 | 40.3 | 1.04 | At least two teeth with PD ≥5 mm and CAL ≥6 mm with BOP at same site | Secure | Serum | Fasting | Yes |

| Author(s)  Year | Country | Study design | Sample size | %Males | Males ratio (PD:C) | Mean age (year) | Age Ratio (PD:C) | Diagnostic Criteria for PD | Diagnostic thresholds (Secure or  Insecure) | Blood collection (serum or  Plasma) | Blood test  (fasting or  non-fasting) | BMI control |
| --- | --- | --- | --- | --- | --- | --- | --- | --- | --- | --- | --- | --- |
| Lobao et al.  (2019) | Brazil | Intervention | 62 | 38.7 | 1.04 | 40.4 | 1.04 | at least two teeth with clinical attachment level ≥6 mm and probing depth ≥5 mm in one or more sites | Secure | Serum | Fasting | NO |
| Loo et al.  (2012) | China | Case-Control | 1290 | 62.6 | 0.92 | 44.9 | 1.15 | Based on 1999 AAP classification: PD >5 mm, CAL >4 mm, gingival recession, tooth mobility | Secure | Serum | NI | NI |
| Marcaccini et al.  (2009) | Brazil | Case-Control | 45 | 33.3 | 1.2 | 43.97 | 1.05 | ≥2 teeth with PD ≥5 mm, CAL ≥6 mm, radiographic bone loss | Secure | Plasma | Fasting | NI |
| Miranda  et al.  (2019) | Brazil | Cross-sectional | 51 | 31 | 1.07 | 52.2 | 1.02 | >30% of sites with PD and CAL ≥4 mm + BOP, ≥6 teeth with ≥1 site showing PD and CAL ≥5 mm and BOP | Secure | Serum | Fasting | Yes |
| Miyazawa  et al.  (2012) | Japan | Case-Control | 70 | 41.4 | 0.61 | 49.59 | 1.13 | Moderate (<4 interproximal sites with ≥50% bone loss) to advanced (≥7 interproximal sites with ≥50% bone loss) periodontitis | Secure | Serum | NI | Yes |
| Nakajima  et al.  (2010) | Japan | Case-Control | 118 | 41.5 | 0.81 | 48.5 | 1.01 | Moderate to advanced periodontitis: CAL (mean 4.5 mm), mean bone loss 38.9%, full-mouth PD, CAL, and radiographic assessment | Secure | Serum | NI | NI |
| Önder et al. (2023) | Turkey | Cross-sectional | 48 | 47.9 | 1.56 | 39.8 | 1.06 | ≥5 mm interdental attachment loss at worst site per tooth | Secure | Serum | NI | No |
| Rabelo et al. (2021) | Brazil | Interventional | 30 | 53.3 | 0.60 | 47.9 | 1.11 | ≥10 teeth CAL ≥5 mm, | Secure | Serum | NI | No |
| Şengül et al. (2023) | Turkey | Cross-sectional | 45 | 37.8 | 1.29 | 37.2 | 1.16 | Stage III grade B(based on 2018 AAP/EFP classification | Secure | Serum | NI | NI |
| Tian et al.  (2013) | China | Case-Control | 654 | 28.7 | 7.33 | 39.83 | 1.46 | CAL >4 mm, PD >5 mm, gingival recession, tooth mobility (based on 1999 AAP classification) | Secure | Plasma | NI | NI |

**Table S2.** Continued

| Author(s)  Year | Country | Study design | Sample size | %Males | Males ratio (PD:C) | Mean age (year) | Age Ratio (PD:C) | Diagnostic Criteria for PD | Diagnostic thresholds (Secure or  Insecure) | Blood collection (serum or  Plasma) | Blood test  (fasting or  non-fasting) | BMI control |
| --- | --- | --- | --- | --- | --- | --- | --- | --- | --- | --- | --- | --- |
| Turkmen  et al.  (2023) | Turkey | Case-Control | 40 | 50 | 1.22 | 37.62 | 1.2 | Interdental CAL ≥5 mm, PD ≥6 mm at ≥2 non-adjacent teeth, bone loss to middle/apical third, ≥30% of teeth affected, ≤4 teeth lost due to periodontitis; Grade B by RBL/age ratio 0.25–1.0 | Secure | Serum | Fasting | Yes |
| Wohlfeil  et al.  (2012) | Germany | Case-Control | 61 | 54.1 | 1.37 | 40.05 | 1.92 | PPD ≥3.6 mm + PAL-V ≥5 mm at >30% of sites; PPD ≥7 mm at ≥4 sites; age >35 years | Secure | Serum | Non-fasting | Yes |
| Yamazaki  et al.  (2005) | Japan | Case-Control | 47 | 40.4 | 0.86 | 42.1 | 0.92 | Moderate to advanced periodontitis defined by PPD, CAL, and radiographic bone loss measured at 6 sites per tooth | Secure | Serum | NI | NI |
| Yilmaz et al. (2022) | Turkey | Cross-sectional | 40 | 50.0 | 1.0 | 39.6 | 1.11 | Stage III grade B(based on 2018 AAP/EFP classification | Secure | Serum | Fasting | NI |
| Zimmermann et al.  (2005) | Brazil | Cross-sectional | 78 | 26.9 | 1.2 | 45.4 | 0.90 | ≥30% sites with PD & CAL ≥4 mm + ≥4 teeth with PD & CAL ≥5 mm | Secure | Serum | Fasting | Yes |

**Table S2.** Continued

Abbreviations: PD, probing depth; PPD, probing pocket depth; CAL, clinical attachment level; BOP, bleeding on probing; GI, gingival index; BMI, body

mass index; NI, not informed.

**Table S3.** Characteristics of mouse studies included in the systematic review and meta-analysis of circulating IL-6 levels.

| Author(s)  Year | Study design | Animal model | Sample size (Control / Experimental) | Control | Induction methods | Induction period | Periodontitis evaluation method | Blood collection (serum or  Plasma) |
| --- | --- | --- | --- | --- | --- | --- | --- | --- |
| Ligature | |  |  |  |  |  |  |  |
| Furutama et al.  (2020) | Experimental study | Female  C57BL/6J mice  8–12 weeks old | 9 / 11 | Healthy mice  without ligation | Ligation (5-0 silk) | 1 week | gingival cytokine | Serum |
| Furutama et al.  (2020) | Experimental study | Female  C57BL/6J mice  8–12 weeks old | 9 / 7 | Healthy mice  without ligation | Ligation (5-0 silk) | 4 weeks | gingival cytokine | Serum |
| Furutama et al.  (2020) | Experimental study | Female  C57BL/6J mice  8–12 weeks old | 9 / 8 | Healthy mice  without ligation | Ligation (5-0 silk) | 8 weeks | gingival cytokine | Serum |
| Han et al.  (2021) | Experimental study | Male  C57BL/6 mice  8 weeks old | 10 / 10 | Healthy mice  without ligation | Ligation (5-0 silk) | 10 days | Alveolar Bone loss evaluated by μCT and Histology | Serum |
| Han  et al.  (2023) | Experimental study | Male  C57BL/6 mice  8 weeks old | 10 / 10 | Healthy mice  without ligation | Ligation (5-0 silk) | 10 days | Histology | Serum |
| Kim et al.  (2023) | Experimental study | Male  C57BL/6J mice  6 weeks old | 5 / 7 | Healthy mice  without ligation | Ligation (6-0 silk) | 3 weeks | CEJ–ABC distance, histology | Serum |
| Li et al.  (2021) | Experimental study | Male  C57BL/6J mice  7 weeks old | 7 / 7 | Healthy mice  without ligation | Ligation (5-0 silk) | 6 weeks | CEJ–ABC distance and histology | Serum |
| Marruganti  et al.  (2024) | Experimental study | Male  C57BL/6J mice  8–12 weeks old | 8 / 8 | Healthy mice  without ligation | Ligation (5-0 silk) | 2 weeks | CEJ–ABC distance and histology | Plasma |
| Matsuda et al.  (2016) | Experimental study | Male  C57BL/6 mice  6 weeks old | 8 / 8 | Healthy mice  without ligation | Ligation (5-0 silk) | 2 weeks | CEJ–ABC distance, histology, gingival cytokine | Serum |
| Xue et al.  (2020) | Experimental study | Male  C57BL/6J mice  12 weeks old | 8 / 8 | Healthy mice  without ligation | Ligation (5-0 silk) | 12 months | CEJ–ABC distance, histology, gingival cytokine | Serum |
| Yang et al.  (2025) | Experimental study | Male  C57BL/6J mice  6 weeks old | 6 / 6 | Healthy mice  without ligation | Ligation (5-0 silk) | 4 weeks | CEJ–ABC distance, histology, Trap staining, gingival cytokine | Serum |

**Table S3.** Continued

| Author(s)  Year | Study design | Animal model | Sample size (Control / Experimental) | Control | Induction methods | Induction period | Periodontitis evaluation method | Blood collection (serum or  Plasma) |
| --- | --- | --- | --- | --- | --- | --- | --- | --- |
| Inoculation | |  | | | | | | |
| Kang et al. (2022) | Experimental study | Male  C57BL/6 mice  6 weeks old | 6 / 6 | PBS + 2% CMC | Oral gavage of *P. gingivalis* | 22 weeks | CEJ–ABC distance, histology, gingival cytokine | Serum |
| Queiroz et al. (2012) | Experimental study | Male  C57BL/6 mice  6 weeks old | 5 /5 | PBS + 1.5% CMC | Oral gavage of  *A. actinomycetemcomitans* | 45 days | Area of alveolar bone loss, TRAP staining | Serum |
| Tamura et al. (2022) | Experimental study | Male  C57BL/6J mice  8 weeks old | 6 / 6 | 2% CMC | Oral gavage of *P. gingivalis* | 6 weeks | CEJ–ABC distance and histology | Serum |

Abbreviations: CEJ, cemento-enamel junction; ABC: alveolar bone crest; TRAP, tartrate-resistant acid phosphatase; CMC, carboxymethylcellulose.
